# Metabolic effect of adrenaline infusion in people with type 1 diabetes and healthy individuals

**DOI:** 10.1007/s00125-024-06116-5

**Published:** 2024-03-01

**Authors:** Rui She, Tommi Suvitaival, Henrik U. Andersen, Eva Hommel, Kirsten Nørgaard, Jørgen F. P. Wojtaszewski, Cristina Legido-Quigley, Ulrik Pedersen-Bjergaard

**Affiliations:** 1 Department of Endocrinology and Nephrology, Nordsjællands Hospital, Hillerød, Denmark; 2 Department of Clinical Medicine, University of Copenhagen, Copenhagen, Denmark; 3 Steno Diabetes Center Copenhagen, Herlev, Denmark; 4 Department of Nutrition, Exercise and Sports, University of Copenhagen, Copenhagen, Denmark; 5 Institute of Pharmaceutical Science, King’s College London, London, UK

**Keywords:** Clinical diabetes, Hypoglycaemia, Metabolic physiology in vivo, Metabolomics, Other hormones/action, Pathophysiology/metabolism

## Abstract

**Aims/hypothesis:**

As a result of early loss of the glucagon response, adrenaline is the primary counter-regulatory hormone in type 1 diabetes. Diminished adrenaline responses to hypoglycaemia due to counter-regulatory failure are common in type 1 diabetes, and are probably induced by exposure to recurrent hypoglycaemia, however, the metabolic effects of adrenaline have received less research attention, and also there is conflicting evidence regarding adrenaline sensitivity in type 1 diabetes. Thus, we aimed to investigate the metabolic response to adrenaline and explore whether it is modified by prior exposure to hypoglycaemia.

**Methods:**

Eighteen participants with type 1 diabetes and nine healthy participants underwent a three-step ascending adrenaline infusion during a hyperinsulinaemic–euglycaemic clamp. Continuous glucose monitoring data obtained during the week before the study day were used to assess the extent of hypoglycaemia exposure.

**Results:**

While glucose responses during the clamp were similar between people with type 1 diabetes and healthy participants, plasma concentrations of NEFAs and glycerol only increased in the group with type 1 diabetes (*p*<0.001). Metabolomics revealed an increase in the most common NEFAs (*p*<0.01). Other metabolic responses were generally similar between participants with type 1 diabetes and healthy participants. Exposure to hypoglycaemia was negatively associated with the NEFA response; however, this was not statistically significant.

**Conclusions/interpretation:**

In conclusion, individuals with type 1 diabetes respond with increased lipolysis to adrenaline compared with healthy participants by mobilising the abundant NEFAs in plasma, whereas other metabolic responses were similar. This may suggest that the metabolic sensitivity to adrenaline is altered in a pathway-specific manner in type 1 diabetes.

**Trial registration:**

ClinicalTrials.gov NCT05095259

**Graphical Abstract:**

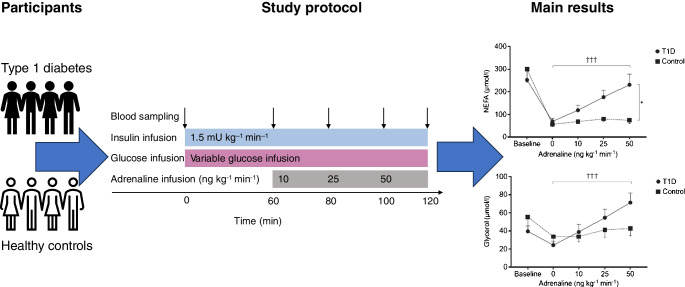

**Supplementary Information:**

The online version contains peer-reviewed but unedited supplementary material available at 10.1007/s00125-024-06116-5.


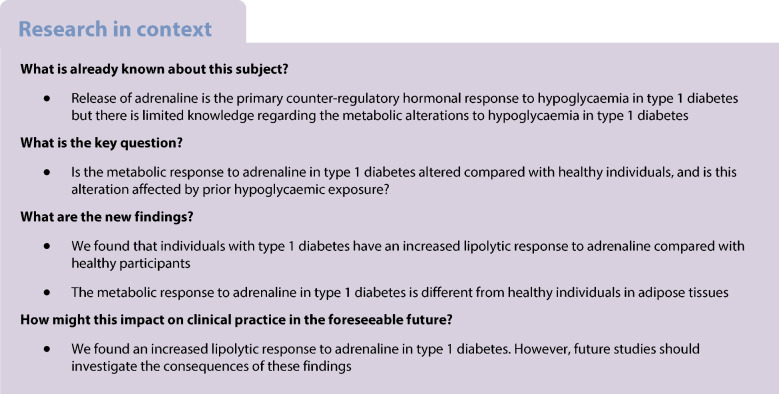


## Introduction

The major limiting factor in achieving optimal glycaemic control in type 1 diabetes is hypoglycaemia, as efforts to lower glucose levels may result in mild or severe episodes of hypoglycaemia [1]. As a result, most individuals with type 1 diabetes experience recurrent hypoglycaemia, with some experiencing it almost daily [2, 3]. A substantial body of evidence suggests that experiencing prior episodes of hypoglycaemia may lead to diminished counter-regulatory responses to future hypoglycaemic events. This results in development of a syndrome characterised by failure of counter-regulatory responses, such as impaired catecholamine responses, as well as reduced awareness of hypoglycaemic symptoms and decreased perception of hypoglycaemic warning signs in people who frequently experience hypoglycaemia [4–6]. This condition confers a considerably increased risk of severe hypoglycaemia, which is the most feared side effect of insulin therapy and a driver of acute morbidity and mortality [7].

As early loss of the glucagon response to hypoglycaemia is common in type 1 diabetes [8], the adrenaline (epinephrine) response emerges as the first-line hormonal defence against developing more severe hypoglycaemia [7, 8]. Adrenaline stimulates both hepatic glycogenolysis and gluconeogenesis through β-adrenergic activation of hepatocytes [9]. Initially, the increase in hepatic glucose production is primarily driven by glycogenolysis, and gluconeogenesis takes over afterwards [9, 10]. Furthermore, lipolysis is highly regulated by β-adrenergic stimulation of adipose tissue [11, 12].

While the effect of recurrent hypoglycaemia in type 1 diabetes on the magnitude of the adrenaline response to hypoglycaemia has been studied extensively [6, 7], knowledge of its impact on metabolic responses to adrenaline is very limited. To test the hypothesis that recent exposure to hypoglycaemia results in changes in the metabolic effects of adrenaline, we compared the metabolic responses to a standardised stepwise adrenaline infusion between healthy people and individuals with type 1 diabetes with varying recent exposure to hypoglycaemia as assessed by continuous glucose monitoring (CGM).

## Methods

This study is an interventional case–control study investigating individuals with type 1 diabetes and healthy individuals. The study was approved by the Regional Committee on Health Research Ethics in the Capital Region of Denmark (H-19031592) and registered at ClinicalTrials.gov (NCT05095259). The study was conducted in accordance with the Declaration of Helsinki.

### Study participants

A total of 27 male and female (self-reported) participants aged 18–70 years were included in this study. Eighteen participants with type 1 diabetes were recruited from the outpatient clinics at Nordsjællands Hospital, Hillerød, Denmark, and the Steno Diabetes Center Copenhagen, Copenhagen, Denmark, and nine healthy participants were recruited through local advertising and advertisement on social media. The diagnosis of type 1 diabetes was clinical and supported by low C-peptide concentration (<20 pmol/l) or absent C-peptide. Our study aimed to include individuals with type 1 diabetes who have varying expected daily exposure to hypoglycaemia. Participants who were already using a real-time CGM were allowed to continue using it during the study. The main exclusion criteria were use of β-blocking or β-activating agents, medical history of cardiovascular diseases, renal impairment or pregnancy at the onset of the study and during participation in the study. No participants were excluded based on sex and our study sample was selected to represent patients with frequent exposure to hypoglycaemia. Written informed consent was obtained. Participants were compensated for loss of work hours incurred through participation in the study.

### Study protocol

The experiment was divided into one screening day and one trial day, at least 7 days apart. The investigation was performed at the Endocrine Research Unit at Nordsjællands Hospital, Hillerød, Denmark. The experiment was explained to the potential participants on the screening day, and inclusion and exclusion criteria were assessed. After obtaining written consent, participants underwent a clinical examination, and screening blood samples were drawn. For the participants with type 1 diabetes, CGMs were used a week before the study day to determine their hypoglycaemic exposure. The first four participants were equipped with a blinded iPro2 with Enlite sensor (Medtronic MiniMed, USA). However, because Medtronic cancelled support for its product, the last 14 participants with type 1 diabetes wore an unblinded Freestyle Libre 2 (Abbott Laboratories, USA). Before the experimental day, participants avoided alcohol intake, caffeine consumption and smoking for at least 24 h. Strenuous physical activity was avoided for 48 h, and participants fasted for at least 7 h prior to the experiment. As exposure to hypoglycaemia was an essential cofactor in the analyses, no measures were taken to avoid hypoglycaemia in participants with type 1 diabetes before the study day. On the experimental day, participants underwent a hyperinsulinaemic–euglycaemic clamp with stepwise adrenaline infusion.

#### Hyperinsulinaemic–euglycaemic clamp

Insulin was infused at a constant rate of 0.75 mU insulin kg^−1^ min^−1^ (Actrapid, Novo Nordisk, Denmark). The plasma glucose (PG) target of 4.0–6.0 mmol/l was maintained by a variable 10% glucose infusion. All participants were held under the hyperinsulinaemic–euglycaemic clamp for 1 h before administering adrenaline.

#### Adrenaline administration

Infusions of adrenaline (Adrenalin ‘SAD’, Amgros, Denmark) were given intravenously continuously throughout the experiment. Adrenaline dissolved in isotonic saline (154 mmol/l NaCl) was administrated at stepwise increasing infusion rates (10, 25 and 50 ng adrenaline kg^−1^ min^−1^), with every step lasting for 20 min. In cases where participants did not achieve euglycaemic targets at the end of a period, the adrenaline infusion rate was increased only after PG levels returned to euglycaemia (4.0–6.0 mmol/l).

### Biochemical analysis

**Fig. 1:**
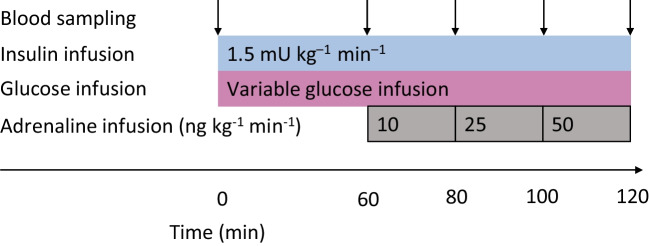
Experimental design. All participants underwent a hyperinsulinaemic–euglycaemic clamp. Blood samples were taken before the clamp (baseline, time 0), 1 h after the start of the clamp (0 ng kg^−1^ min^−1^) and three times during the stepwise adrenaline infusion (10–50 ng kg^−1^ min^−1^). Blood samples for untargeted metabolomics profiling were taken before the initiation of the hyperinsulinaemic–euglycaemic clamp (baseline), 1 h after the start of the clamp and 20 min after the last infusion

During the first hour of the clamp, PG was analysed every 10 min using a YSI 2300 analyser (YSI/Xylem, USA). During the adrenaline infusions, PG was measured every 5 min. Plasma samples for analysis of insulin, glucagon, adrenaline, noradrenaline (norepinephrine), NEFAs and glycerol were acquired before the clamp (baseline), 1 h from the start of the clamp (0 ng kg^−1^ min^−1^) and 20 min after each step of the stepwise adrenaline infusion (10–50 ng kg^−1^ min^−1^) (Fig. 1). All plasma samples were mixed with EDTA and placed in a dry ice box at first, and then stored in a −80°C freezer. ELISA kits were used to analyse insulin (catalogue no. 80-INSHU-E01.1, ALPCO, USA) and glucagon (catalogue no. 10-1271-01, Mercodia, Sweden); these kits use antibodies to determine the insulin and glucagon concentration using a colorimetric endpoint read spectrophotometrically by a SpectraMax iD3 (Molecular Devices, USA). Adrenaline and noradrenaline were quantified using a 2-CAT Plasma ELISA^High Sensitive^ kit (Labor Diagnostika Nord, Germany), which extracts, acylates and enzymatically converts adrenaline and noradrenaline. Glycerol (Randox, UK) and NEFAs (NEFA C kit, Wako Chemicals, Germany) were used to perform an enzymatic transformation of the metabolites, which were measured using a COBAS autoanalyser (Roche, Switzerland). All analyses were performed according to the manufacturers’ instructions.

#### Untargeted metabolomics profiling

Plasma samples for metabolomics were acquired at baseline, 1 h from the start of the clamp and 20 min after the last infusion (50 ng adrenaline kg^−1^ min^−1^) using containers containing heparin. Plasma samples (30 μl) were mixed with 400 μl methanol, 10 μl internal standard mixture succinic acid-d4, glutamic acid-d5, valine-d8 and heptadecanoic acid-d33, Sigma Aldrich, USA), and incubated on ice for 30 min. Samples were then centrifuged (10,000 *g*, 3 min, 4°C), and 180 μl of the filtered extracts were evaporated to dryness and derivatised. A detailed description of the process has been presented previously [13]. A 7250 GC/Q-TOF instrument (Agilent, USA) equipped with a Gerstel MPS autosampler (Gerstel, Germany) was used to analyse the samples. Features from the quantitative ion peak areas of the data were matched against mass spectral libraries (in-house library, Fiehn library [14], GOLM DB [15], GNPS [16], HMDB [17] and MassBank Japan [18]).

### CGM data assessments

CGM data were analysed in accordance with current consensus guidelines from 2017 and 2019 [19, 20]. Hypoglycaemia episodes were defined as those persisting for a minimum of 15 min, and were categorised into two levels. Level 1 was defined as glucose levels below 3.9 mmol/l (<70 mg/dl), and level 2 was defined as glucose levels below 3.0 mmol/l (<54 mg/dl). We used two distinct variables of hypoglycaemia exposure: one based on the time below range (TBR) and the other based on the number of hypoglycaemic episodes per week (HEPW).

### Endpoints and assessments

Primary endpoints were the incremental area under the PG curve (iAUC) as a measurement of glucose production, and plasma levels of NEFAs and glycerol as a measure of lipolysis before and after each step of adrenaline infusion. A secondary endpoint was the untargeted metabolomics profiling of the blood samples taken at baseline, 1 h from the start of the clamp and at the end of the adrenaline infusion.

### Statistical analysis

Endpoints were statistically assessed using linear mixed-effect models. Repeated measurements of PG iAUC, NEFA and glycerol were analysed from the start of the clamp until the end of the trial (0–50 ng kg^−1^ min^−1^). Data from untargeted metabolomics profiling were analysed at baseline, 0 and 50 ng kg^−1^ min^−1^ (Fig. 1). The *p* values for the results from the metabolomics data were adjusted for multiple testing and false discovery rate using the Benjamini–Hochberg method (*p*_BH_) [21].

When analysing PG iAUC, NEFA and glycerol, between-group assessments (type 1 diabetes vs healthy participants) were modelled using intervention, group and group–interaction as fixed effects and participant ID as a random effect and intercept with intervention. As we had not obtained multiple measurements for each participant, the statistical analysis was performed similarly for metabolomics data but without random intercept with intervention. The same models were used to analyse the intervention within both groups. Lastly, TBR and HEPW were used as variables in the model to explore the association with the endpoints. The statistical analyses were performed using SPSS Statistics (IBM, USA) and R software [22].

## Results

### Participant characteristics

The baseline characteristics of the 27 participants are shown in Table 1. The group with type 1 diabetes had a higher proportion of male participants and the participants were older compared with the control group. As anticipated, the mean HbA_1c_ value was higher in the type 1 diabetes group compared with the healthy participants. In addition, participants with type 1 diabetes showed pronounced variations in HbA_1c_ levels, with a median value of 51.0 mmol/mol (IQR 44–62) or 7.0% (6.2–8.0). Eight of the 18 diabetic participants used insulin pumps and 12 used CGM devices before participation. The median value of TBR level 1 was 1.8% (IQR 0.0–6.5). For HEPW level 1, the median value was 5.6 (IQR 0.0–8.1).

**Table 1:** Baseline characteristics

Variable	Diabetic participants (*N*=18)	Healthy participants (*N*=9)
Men	12 (67%)	4 (44%)
Age, years	44.6 (3.7)	35.9 (5.4)
BMI, kg/m^2^	26.6 (1.2)	25.9 (1.8)
HbA_1c_, mmol/mol	56 (4.2)	35 (1.6)
HbA_1c_, %	7.3 (0.38)	5.3 (0.15)
C-peptide, pmol/l	4 (0.0–14.3)	467.0 (364.5–527.0)
Duration of type 1 diabetes, years	20.2 (2.5)	
Hillerød method		
Normal	10 (56%)	
Impaired awareness	4 (22%)	
Unawareness	4 (22%)	
Clarke score		
Normal	9 (50%)	
Unclassifiable	5 (28%)	
Reduced	4 (22%)	
Gold score		
Normal	12 (67%)	
Impaired	6 (33%)	
TBR, level 1, %	1.8 (0.0–6.5)	
TBR, level 2, %	0.0 (0.0–1.6)	
HEPW, level 1	5.6 (0.0–8.1)	
HEPW, level 2	0.0 (0.0–3.0)	
Freestyle Libre 2	14 (78%)	
Insulin pump therapy	8 (44%)	

Values are *n* (%), mean (SEM) or median (IQR). The number of participants using Freestyle Libre 2 is shown; the remaining participants with type 1 diabetes (four) used an iPro2

The Hillerød method [56], Clarke score [5] and Gold score [4] are the most commonly used self-estimated hypoglycaemia awareness questionnaires

### Glucose infusion rate and insulin levels

**Fig. 2:**
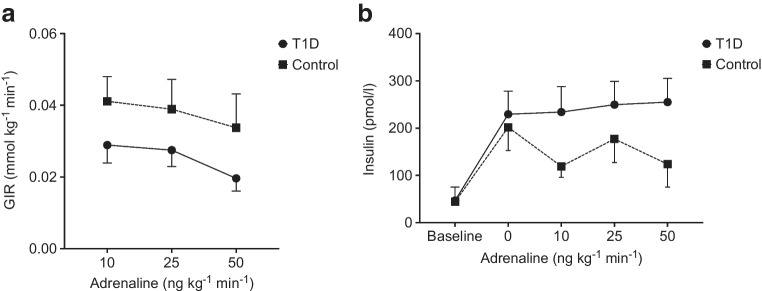
(**a**) Mean glucose infusion rate (GIR) at each step of the adrenaline infusion. (**b**) Mean plasma insulin levels before the hyperinsulinaemic–euglycaemic clamp (baseline), 1 h after the start of the clamp (0 ng kg^−1^ min^−1^) and during the stepwise adrenaline infusion (10–50 ng kg^−1^ min^−1^). Error bars indicate the SEM. Circles with a solid line represent participants with type 1 diabetes; squares with a dotted line represent healthy control participants

Throughout the stepwise adrenaline infusion, the glucose infusion rate did not change significantly in the group with type 1 diabetes (*p*=0.13) or healthy participants (*p*=0.38) (Fig. 2a). Healthy participants required a higher glucose infusion rate to achieve euglycaemia than the group with type 1 diabetes throughout the experiment (*p*=0.044). The difference did not correlate with the adrenaline dose (*p*=0.94). Insulin levels did not alter during the trial in either participants with type 1 diabetes (*p*=0.68) or healthy participants (*p*=0.19) (Fig. 2b). Participants with type 1 diabetes had numerically higher insulin levels than healthy participants. However, the difference was not significant (*p*=0.41), and the insulin concentration did not alter differently to the adrenaline infusion between the groups (*p*=0.091).

### Adrenaline, noradrenaline and glucagon levels

**Fig. 3:**
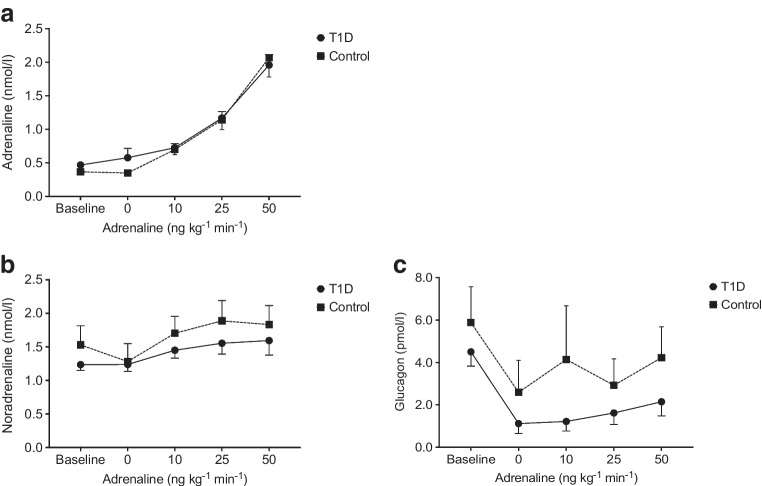
Plasma concentrations of adrenaline (**a**), noradrenaline (**b**) and glucagon (**c**) before the clamp (baseline), 1 h after the start of the clamp (0 ng kg^−1^ min^−1^) and during the stepwise adrenaline infusion (10–50 ng kg^−1^ min^−1^). Error bars indicate the SEM. Circles with a solid line represent participants with type 1 diabetes; squares with a dotted line represent healthy control participants

Plasma adrenaline levels (Fig. 3a) increased significantly and continuously during the infusion of adrenaline in both groups (*p*<0.001), and no difference in adrenaline concentrations between groups was detected (*p*=0.78). Similar increases in plasma noradrenaline levels were observed in the type 1 diabetes and control group (Fig. 3b) but the increase was only significant in the healthy participants (*p*=0.008). When comparing the plasma noradrenaline levels between groups, there was no difference (*p*=0.55), and nor did we observe a difference between the two groups in terms of the noradrenaline responses to the adrenaline infusion (*p*=0.57). The plasma glucagon concentration significantly increased throughout the adrenaline infusion in participants with type 1 diabetes (*p*=0.016) but not in the control group (*p*=0.067) (Fig. 3c). However, the between-group comparison showed no significant difference in glucagon concentration (*p*=0.16) or in the response of glucagon concentrations to adrenaline infusion (*p*=0.95).

### Plasma glucose iAUC

**Fig. 4:**
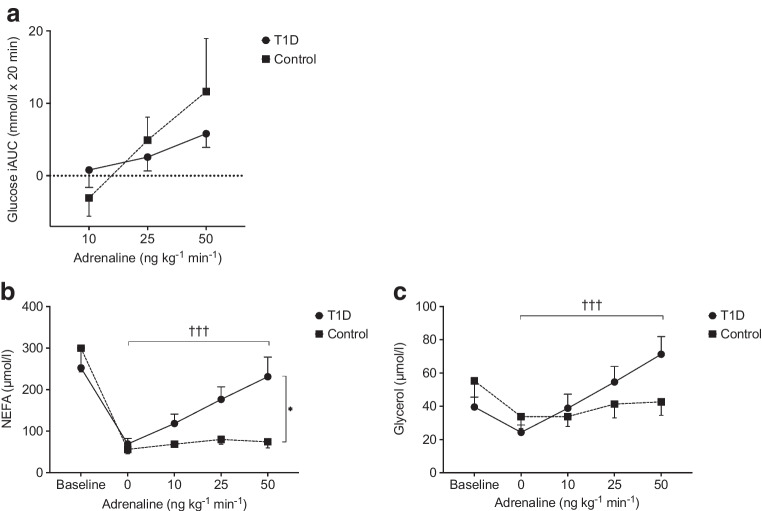
(**a**) PG iAUC at each step of the adrenaline infusion. (**b**, **c**) Plasma NEFA (**b**) and glycerol (**c**) levels before the clamp (baseline), 1 h after the start of the clamp (0 ng kg^−1^ min^−1^) and during the stepwise adrenaline infusion (10–50 ng kg^−1^ min^−1^). Error bars indicate the SEM. Circles with a solid line represent participants with type 1 diabetes; squares with a dotted line represent healthy control participants. Between-group analysis, *p≤0.05; type 1 diabetes within-group analysis, ^†††^*p*≤0.001 µmol/l

The mean PG iAUC tended to increase in both groups during the stepwise intervention (Fig. 4a). However, the elevations were not significant (type 1 diabetes, *p*=0.21 and control, *p*=0.15). The mean iAUC for the group with type 1 diabetes did not differ from healthy participants (*p*=0.46).

### Plasma NEFA and glycerol levels

Both groups showed a pronounced reduction in NEFA and glycerol levels between baseline and before the initiation of the adrenaline infusion (0 ng kg^-1^ min^-1^ adrenaline) following the insulin infusion (Fig. 4b,c). In the control group, the decrement in glycerol was statistically insignificant (paired sample *t* test, *p*=0.13). Independent analysis of the groups showed that NEFA levels increased significantly in the group with type 1 diabetes (*p*<0.001) but remained unaltered in healthy participants during the adrenaline infusions (*p*=0.066). When comparing the groups, the incremental NEFA response to adrenaline in type 1 diabetes was greater than that in healthy participants (*p*=0.029). In the type 1 diabetes group, glycerol levels increased significantly in response to the intervention (*p*<0.001), in contrast to the control group, which did not show a significant response (*p*=0.24). However, when comparing the two groups, no significant differences were detected (*p*=0.075). Post hoc sensitivity analyses adjusting for insulin sensitivity, the ratio of the *M* value and corresponding insulin concentration (M/I ratio) calculated from the study, and age were performed and did not alter the differences found between participants with type 1 diabetes and healthy participants (see electronic supplementary material [ESM] Table 1).

### Untargeted metabolomics profiling

**Fig. 5:**
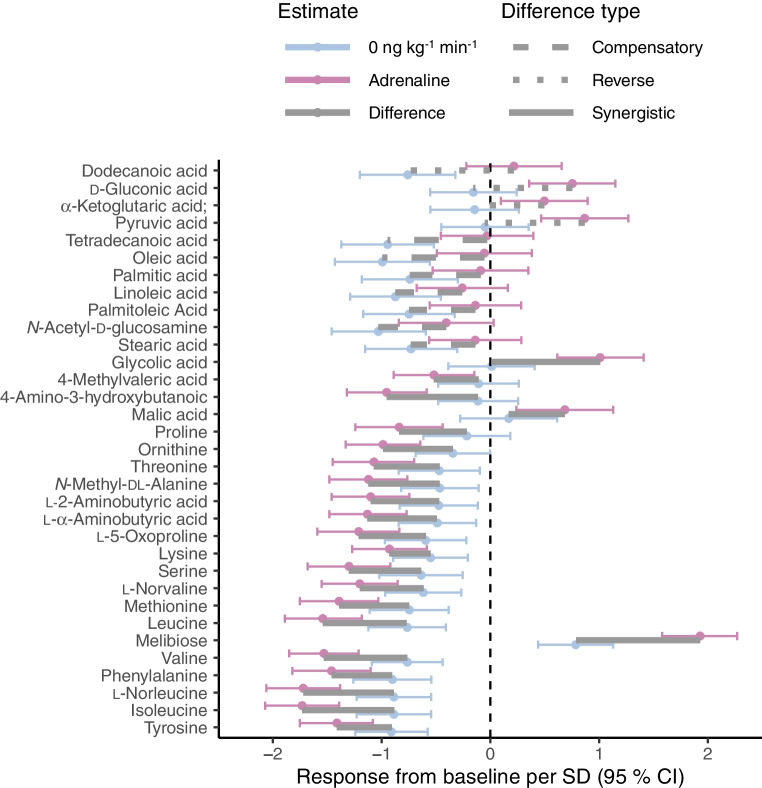
Forest plot of the metabolites that responded to adrenaline in participants with type 1 diabetes. Blue points and error bars indicate the response to the hyperinsulinaemic–euglycaemic clamp (from baseline to 0 ng kg^−1^ min^−1^). Purple points and error bars indicate the metabolic response to the whole stepwise adrenaline infusion protocol (the total response from baseline to 20 min after the last infusion of 50 ng kg^−1^ min^−1^). Grey bars indicate the difference between the two metabolic responses (i.e. how metabolite levels respond to adrenaline from the hyperinsulinaemic state). ‘Synergistic’ response indicates a metabolic response to the clamp and the adrenaline infusion in the same direction (solid grey bar). ‘Reverse’ response indicates a response that goes in the opposite direction (dotted grey bar). ‘Compensatory’ response indicates a reverse response without crossing the baseline concentration (dashed grey bar). Estimates are standardised according to the standard deviation of the metabolite (i.e. *z* scores). Error bars indicate 95% CI

Untargeted metabolomics profiling identified a total of 162 metabolites. Of these, 33 showed changes following stepwise adrenaline infusion (Fig. 5). During the first hour of the hyperinsulinaemic–euglycaemic clamp, levels of all 33 metabolites except melibiose decreased or remained unaltered. After adrenaline infusion, the levels of ten amino acids decreased further, and the levels of many of the abundant fatty acids increased after adrenaline infusion. Both saturated and unsaturated NEFAs with carbon chains of lengths between 12–18 increased after adrenaline infusion, most notably oleic acid, palmitic acid, stearic acid and linoleic acid (*p*_BH_ <0.01). The concentrations of other important energy metabolites also changed: levels of pyruvic, α-ketoglutaric and malic acid increased while ornithine levels decreased. The plasma levels of melibiose, which increased during the first hour of the hyperinsulinaemic–euglycaemic clamp, increased further after adrenaline infusion. All mentioned metabolites showed significant alteration after adjusting for multiple testing (*p*_BH_ <0.05). In the between-group analysis, raw *p* values before adjusting for multiple testing showed a total of 11 metabolites for which responses differed between type 1 diabetes and healthy participants, including the fatty acids palmitic, oleic and linoleic acid, which had a greater incremental response in the group with type 1 diabetes. After adjusting for multiple testing, the differences were not significant (ESM Table 2).

### Impact of hypoglycaemia exposure in type 1 diabetes

In the type 1 diabetes group, TBR levels 1 and 2 were not correlated with the elevation in PG iAUC (*p*>0.50), and TBR did not affect the incremental responses in plasma levels of NEFA and glycerol to adrenaline (*p*>0.33). The same was true for HEPW levels 1 and 2. Analysing the same four hypoglycaemic variables on the metabolomics data showed that, before adjusting for multiple testing, between 6 and 23 metabolite responses were associated with hypoglycaemia exposure. Notably, NEFAs whose levels increased in response to adrenaline in the type 1 diabetes group (palmitic acid, stearic acid, oleic acid and linoleic acid) were negatively correlated with TBR and HEPW at level 2 but not level 1. Thus, hypoglycaemic exposure under 3.0 mmol/l was negatively associated with the increase in NEFA levels in type 1 diabetes. However, when adjusted for multiple testing, these alterations were not significant (ESM Table 3)*.* To examine the impact of switching to Freestyle Libre 2 on metabolic outcomes, we performed a sensitivity analysis that excluded the four participants on iPro2 with the Enlite sensor. This did not result in any change in the effect of hypoglycaemic exposure (data not shown).

### Cardiovascular responses

Heart rate was measured during the adrenaline infusion. Heart rate increased in both groups during the intervention, but no differences were observed between groups, nor did we find any association with hypoglycaemic exposure (data not shown).

## Discussion

The metabolic response to adrenaline infusion showed notable differences between individuals with type 1 diabetes and healthy participants in this study. Specifically, concentrations of the most abundant NEFAs significantly increased in individuals with type 1 diabetes, but no such increase was observed in the control group. A similar trend was observed for glycerol, suggesting that adrenaline stimulates lipolysis in individuals with type 1 diabetes but not in healthy participants. Previous studies evaluating lipolysis in type 1 diabetes have shown diverging results. One study showed no increase in lipolysis in individuals with type 1 diabetes compared with healthy participants following experimental adrenaline infusion [23]. In contrast, another study observed reduced lipolysis in response to hypoglycaemia in individuals with type 1 diabetes compared with healthy participants [24]. However, the latter finding was most likely due to a diminished adrenaline response in the type 1 diabetes group. Several studies have shown increased lipolysis during adrenaline infusion [25–27], and a seemingly increased β-adrenergic response in adipose tissues during hypoglycaemia in type 1 diabetes [28–30]. Our findings support the latter result: because, during the last infusion (50 ng adrenaline kg^−1^ min^−1^), our participants showed adrenaline concentrations resembling levels induced by hypoglycaemia [24, 27, 29, 31], our results reflect metabolic changes expected to occur during the hypoglycaemia experienced in daily life in individuals with type 1 diabetes.

Furthermore, metabolomic profiling in type 1 diabetes revealed that the increase in NEFAs is dominated by the most common circulating NEFAs in plasma: palmitic acid, stearic acid, oleic acid and linoleic acid [32, 33]. We also observed an increase in PG due to the adrenaline infusion, as expected due to its stimulation of hepatic glycogenolysis [10, 34] and inhibition of splanchnic and peripheral glucose uptake [35]. However, we did not observe a difference in glucose production between type 1 diabetes and healthy participants. Our results indicate that the higher sensitivity to adrenaline in type 1 diabetes is pathway-specific, as observed previously [36]. The increase in lipolysis may be an alternative metabolic mechanism to support energy metabolism during hypoglycaemia.

Metabolomic profiling also revealed decreased levels of ketogenic and glucogenic amino acids. Previous studies have found similar results [37, 38]. Studies examining plasma amino acid kinetics in response to adrenaline infusion have demonstrated that adrenaline transiently stimulates increased disappearance of circulatory amino acids, but its effect diminishes over time [39, 40]. The mechanism underlying this effect remains unclear and requires further investigation. Interestingly, our metabolomics analysis also revealed that the levels of three intermediates of the citric acid cycle were elevated after adrenaline infusion: pyruvic acid (pyruvate), α-ketoglutaric acid (α-ketoglutarate) and malic acid (malate). Furthermore, levels of ornithine, a critical intermediate in the urea cycle, decreased [41, 42]. The citric acid cycle is crucial in ATP production by the cells, using products of glycolysis, fatty acid β-oxidation and ketogenic breakdown of amino acids. The urea cycle is important in the degradation of amino acids into urea but also in the production of several intermediates in the citric acid cycle. Previous work has found elevated levels of intermediates of the citric acid cycle in skeletal muscle when exposed to adrenaline [43]. These findings suggest a role of adrenaline in supporting energy metabolism by facilitating the citric acid cycle through provision of intermediates, and our results support this suggestion. Lastly, levels of melibiose were observed to increase after the adrenaline intervention. Because of its properties as a disaccharide that is not produced nor metabolised by humans, previous studies have used melibiose to assess intestinal permeability [44, 45]. Thus, increased melibiose levels suggest increased intestinal permeability due to adrenaline infusion. Previous animal studies have found that adrenaline increases intestinal absorption [46, 47]. However, to our knowledge, no human studies have explored this topic.

Glucagon concentrations were numerically lower in the participants with type 1 diabetes compared with healthy participants, but the difference was not statistically significant. In our study, adrenaline stimulated glucagon secretion in individuals with type 1 diabetes as shown previously [48, 49]. However, interestingly, the insulin clamp suppressed the stimulatory effect of adrenaline on glucagon secretion to below basal levels (Fig. 3). One previous study obtained similar findings when applying a 2 h adrenaline infusion during a hyperinsulinaemic–euglycaemic clamp [36]. Collectively, these results may indicate that insulin not only exerts a suppressive influence on alpha cell activity but also prevails over the stimulatory impact of adrenaline under physiological conditions.

Assessing hypoglycaemic exposure’s impact on the metabolite alterations from the metabolomics analysis to the adrenaline infusion indicates that participants with type 1 diabetes and increased exposure to hypoglycaemia under 3.0 mmol/l (<54 mg/dl) have a reduced response for several of the most common circulatory NEFAs. This association, which disappeared in the adjusted analyses, appears to be maladaptive rather than adaptive in supporting metabolism during hypoglycaemia in type 1 diabetes. However, the decrease may also be caused by an increased circulatory disappearance and metabolisation of the NEFAs. In one study, experimental hypoglycaemia was induced before adrenaline infusion and did not result in any altered response in lipolysis and glucose metabolism [27]. Previous studies have associated hypoglycaemia exposure with reduced β-adrenergic sensitivity in type 1 diabetes [50, 51]. However, in both studies, cardiopulmonary rather than metabolic outcomes were used as a measure of β-adrenergic sensitivity. We observed an increase in heart rate in the type 1 diabetes group and healthy participants, but did not observe a difference between the groups, nor did we find any association with hypoglycaemic exposure (data not shown).

A strength of this study is the use of a three-step infusion of adrenaline to explore its metabolic properties over the physiological range of plasma levels. Very few studies have used this approach. One previous study used a dose–response intervention to investigate glucose and amino acid responses in healthy participants by applying adrenaline infusion rates between 7.0 and 30 ng adrenaline kg^−1^ min^−1^ to avoid adrenaline levels that corresponded to a severe hypoglycaemic response [39]. They found that alterations in glucose and amino acids were already observed during the low infusion rate. Other studies have used adrenaline infusion rates ranging from 10 ng adrenaline kg^−1^ min^−1^ [52, 53] to 60 ng adrenaline kg^−1^ min^−1^ [36, 43]. We selected infusion rates of 10, 25 and 50 ng adrenaline kg^−1^ min^−1^ to assess metabolic responses during adrenaline levels comparable to those of a hypoglycaemic counter-regulatory response in order to investigate responses that may be similar to those that individuals with type 1 diabetes would encounter in their daily life. Another strength of the study is the use of comprehensive metabolic assessments to explore potential new metabolic alterations.

Our study has limitations. All participants underwent a hyperinsulinaemic–euglycaemic clamp to ensure that all participants with type 1 diabetes remained within the glycaemic target during the experiment. This resulted in elevated levels of plasma insulin throughout the study, which may have affected the outcomes. This limitation mostly affects our interpretation of the decreases in metabolite levels found using metabolomics profiling (Fig. 5), which are dominated by amino acids. However, previous studies found similar decreases in plasma amino acid concentrations in response to adrenaline [37, 38]. Furthermore, we used the first hour to reach a steady state between the insulin infusion and glucose infusion rate [54], thereby reducing the effect of insulin on metabolism during the adrenaline infusion.

Plasma insulin levels were numerically higher in the type 1 diabetes group compared with the healthy participants. Combined with the control group’s higher glucose infusion rate throughout the study, this is indicative of reduced insulin sensitivity in the type 1 diabetes group [55], which is a well-known phenomenon, as shown in other studies [27, 29]. This may affect our observed differences in levels of NEFAs and glycerol. However, sensitivity analyses adjusting for insulin sensitivity and age did not alter our main findings (ESM Tables 1 and 2). We did not conduct a dedicated sex analysis of our outcomes in this study as the number of participants included in the study did not provide sufficient statistical power to conduct a robust analysis stratified by sex.

In conclusion, we observed an increased lipolytic response to adrenaline infusion in individuals with type 1 diabetes compared with healthy participants. The responses included increases in the most abundant fatty acids. They may indicate an adaptation to hypoglycaemic exposure by preserving fuel supply during future hypoglycaemic events in individuals with type 1 diabetes. Additionally, our study showed that the stimulatory effect of adrenaline on glucagon secretion in physiological levels does not prevail over the suppressive effect of insulin, which may be highly relevant when individuals with type 1 diabetes experience insulin-induced hypoglycaemia.

### Supplementary Information

Below is the link to the electronic supplementary material.

Supplementary file1 (PDF 484 KB)

## Data Availability

All data supporting the findings of this study are available within the paper and ESM. Additional datasets are available from the corresponding author upon reasonable request.

## Abbreviations

CGM: Continuous glucose monitoring
GIR: Glucose infusion rate
HEPW: Hypoglycaemic events per week
iAUC: Incremental AUC
M/I ratio: Ratio of the *M* value and plasma insulin concentration
*p*_BH_: Benjamini–Hochberg adjusted *p* value
PG: Plasma glucose
TBR: Time below range
