# Supplementary material for: Metabolic effect of adrenaline infusion in people with type 1 diabetes and healthy individuals
Source: Diabetologia. 2024 Mar 1;67(6):1095–106. doi: 10.1007/s00125-024-06116-5 (PMC11058679; doi:10.1007/s00125-024-06116-5)
Supplement: Supplementary file 1 — Supplementary file1 (PDF 484 KB) [file 125_2024_6116_MOESM1_ESM.pdf]

Electronic Supplementary Material Table 1a: Linear mixed model comparison analyses of the stepwise adrenaline infusion in the participants with type 1 diabetes and healthy controls. Linear mixed model with and without group-intervention interaction were performed on GIR and the hormonal parameters, while only linear mixed model with group-intervention interaction were performed on the metabolic outcomes.

	Parameter	Coefficient	95% Confidence interval		P
			Lower	Upper	
GIR	Group	-135.80	-267.48	-4.12	0.044
	Group – Adrenaline	-0.071	-3.01	2.87	0.94
Insulin	Group	8.59	-12.45	29.63	0.41
	Group – Adrenaline	0.21	-0.045	0.47	0.10
Adrenaline	Group	0.043	-0.27	0.35	0.78
	Group – Adrenaline	-0.0058	-0.018	0.0062	0.70
Noradrenaline	Group	-0.14	-0.68	0.39	0.55
	Group – Adrenaline	-0.0030	-0.012	0.0062	0.57
Glucagon	Group	-1.95	-4.71	0.81	0.16
	Group – Adrenaline	0.0011	-0.033	0.036	0.95
Metabolic Outcome					
iAUC	Group – Adrenaline	-0.10	-0.40	0.20	0.46
NEFA	Group – Adrenaline	3.28	0.51	6.04	0.029
Glycerol	Group – Adrenaline	0.72	-0.074	1.51	0.075

Electronic Supplementary Material Table 1b: Linear mixed model comparison analyses of the stepwise adrenaline infusion in the participants with type 1 diabetes and healthy controls, adjusted for M/I ratio and age. Linear mixed model with and without group-intervention interaction were performed on GIR and the hormonal parameters, while only linear mixed model with group-intervention interaction were performed on the metabolic outcomes.

	Parameter	Coefficient	95% Confidence interval		P
			Lower	Upper	
GIR	Group	-154.42	-293.39	-15.44	0.031
	Group – Adrenaline	0.44	-2.40	3.29	0.75
Insulin	Group	11.31	-4.30	26.93	0.15
	Group – Adrenaline	0.22	-0.034	0.47	0.088
Adrenaline	Group	0.015	-0.29	0.32	0.92
	Group – Adrenaline	-0.0076	-0.020	0.0043	0.20
Noradrenaline	Group	-0.35	-0.81	0.11	0.13
	Group – Adrenaline	-0.0024	-0.012	0.0072	0.61
Glucagon	Group	-1.95	-4.88	0.98	0.18
	Group – Adrenaline	0.0011	-0.035	0.037	0.95
Metabolic Outcome					
iAUC	Group – Adrenaline	-0.10	-0.39	0.19	0.49
NEFA	Group – Adrenaline	3.28	0.51	6.04	0.022
Glycerol	Group – Adrenaline	0.78	0.26	1.30	0.005

Electronic Supplementary Material Table 2a: Metabolite changes to the adrenaline infusions in participants with type 1 diabetes using untargeted metabolomics profiling. Metabolomics data before and after the completion of the stepwise adrenaline infusions were assessed using linear mixed-effects model. Data are presented as a coefficient with confidence interval. Raw P-values are shown and the adjusted P-values using Benjamini-Hochberg.

Metabolites	Coefficient	95% Confidence interval		P	P_{BH}
		Lower	Upper		
Melibiose	1.1400	0.7970	1.49000	3.61e-09	1.77e-07
Glycolic acid	1.0000	0.6050	1.40000	2.77e-06	6.74e-05
Isoleucine	-0.8470	-1.1900	-0.50500	4.13e-06	6.74e-05
L-Norleucine	-0.8330	-1.1800	-0.49000	5.85e-06	7.17e-05
Valine	-0.7670	-1.0900	-0.44400	9.40e-06	9.21e-05
Pyruvic acid	0.9170	0.5160	1.32000	1.79e-05	1.11e-04
4-amino-3-hydroxyButanoic acid	-0.8400	-1.2100	-0.47200	1.81e-05	1.11e-04
D-Gluconic acid	0.9110	0.5120	1.31000	1.81e-05	1.11e-04
Dodecanoic acid	0.9770	0.5390	1.41000	2.75e-05	1.50e-04
Leucine	-0.7710	-1.1300	-0.41500	4.30e-05	2.11e-04
Tetradecanoic acid	0.9150	0.4900	1.34000	4.90e-05	2.12e-04
Oleic acid	0.9370	0.5000	1.37000	5.19e-05	2.12e-04
Ornithine	-0.6420	-0.9850	-0.30000	3.42e-04	1.29e-03
N-Methyl-DL-Alanine	-0.6550	-1.0100	-0.29900	4.42e-04	1.55e-03
L-α-Aminobutyric acid	-0.6400	-0.9970	-0.28200	6.13e-04	1.97e-03
Methionine	-0.6440	-1.0100	-0.28300	6.43e-04	1.97e-03
L-2-Aminobutyric acid	-0.6300	-0.9900	-0.26900	8.08e-04	2.20e-03
Serine	-0.6640	-1.0400	-0.28400	8.10e-04	2.20e-03
L-Norvaline	-0.5870	-0.9380	-0.23600	1.29e-03	3.34e-03
L-5-Oxoproline	-0.6180	-0.9930	-0.24300	1.53e-03	3.75e-03
Threonine	-0.6050	-0.9780	-0.23200	1.78e-03	4.16e-03
α-Ketoglutaric acid	0.6420	0.2390	1.04000	2.15e-03	4.79e-03
Phenylalanine	-0.5580	-0.9150	-0.20000	2.65e-03	5.50e-03
Proline	-0.6220	-1.0200	-0.22200	2.69e-03	5.50e-03
Tyrosine	-0.5040	-0.8370	-0.17100	3.43e-03	6.73e-03
Palmitic acid	0.6500	0.2090	1.09000	4.31e-03	8.13e-03
Linoleic acid	0.6150	0.1960	1.03000	4.51e-03	8.18e-03
Palmitoleic acid	0.6100	0.1900	1.03000	4.94e-03	8.64e-03
N-Acetyl-D-glucosamine	0.6240	0.1890	1.06000	5.47e-03	9.24e-03
Stearic acid	0.5920	0.1680	1.02000	6.72e-03	1.10e-02
Malic acid	0.5170	0.0710	0.96300	2.36e-02	3.73e-02
4-Methylvaleric acid	-0.4100	-0.7820	-0.03820	3.11e-02	4.63e-02
Lysine	-0.3790	-0.7220	-0.03500	3.12e-02	4.63e-02
Deconaic acid	0.4390	0.0306	0.84800	3.54e-02	5.11e-02
Glycine	-0.3390	-0.6860	0.00904	5.61e-02	7.86e-02
Alanine	-0.3180	-0.6890	0.05340	9.24e-02	1.26e-01
Aspartic acid	0.3210	-0.0854	0.72700	1.20e-01	1.59e-01
Maltose	-0.3290	-0.7960	0.13900	1.66e-01	2.14e-01
Glutamine	-0.2200	-0.5940	0.15500	2.47e-01	3.10e-01
Glutamic acid	-0.1970	-0.5750	0.18200	3.04e-01	3.73e-01
L-Histidine	-0.1760	-0.5350	0.18200	3.30e-01	3.95e-01
L-Tryptophan	-0.1800	-0.6340	0.27400	4.33e-01	5.06e-01
D-(+)-Xylose	-0.0739	-0.4420	0.29500	6.91e-01	7.88e-01
3-Indoleacetic acid	-0.0473	-0.4080	0.31300	7.95e-01	8.70e-01
Cysteine	0.0510	-0.3450	0.44800	7.99e-01	8.70e-01
Glyoxylic acid	-0.0361	-0.4170	0.34500	8.51e-01	8.91e-01
D-Galactose	-0.0400	-0.4730	0.39300	8.55e-01	8.91e-01
Malonic acid	0.0335	-0.4130	0.47900	8.82e-01	9.00e-01
3-Hydroxybutyric acid	0.0176	-0.4220	0.45700	9.37e-01	9.37e-01

Electronic Supplementary Material Table 2b: Metabolite changes to the adrenaline infusions in participants with type 1 diabetes using untargeted metabolomics profiling adjusted for M/I ratio and age. Metabolomics data before and after the completion of the stepwise adrenaline infusions were assessed using linear mixed-effects model. Data are presented as a coefficient with confidence interval. Raw P-values are shown and the adjusted P-values using Benjamini-Hochberg.

Metabolites	Coefficient	95% Confidence interval		P	P_{BH}
		Lower	Upper		
Melibiose	1.1300	0.7640	1.5000	4.60e-08	2.16e-06
Isoleucine	-0.8830	-1.2400	-0.5260	5.07e-06	1.06e-04
L-Norleucine	-0.8710	-1.2300	-0.5130	6.76e-06	1.06e-04
Glycolic acid	0.9380	0.5150	1.3600	3.40e-05	3.17e-04
Oleic acid	1.0200	0.5480	1.4800	4.70e-05	3.17e-04
Dodecanoic acid	1.0200	0.5500	1.4900	4.73e-05	3.17e-04
Leucine	-0.8100	-1.1800	-0.4360	4.95e-05	3.17e-04
Valine	-0.7370	-1.0800	-0.3920	6.02e-05	3.17e-04
Tetradecanoic acid	0.9660	0.5100	1.4200	6.80e-05	3.17e-04
4-amino-3-hydroxyButanoic acid	-0.8170	-1.2000	-0.4320	6.81e-05	3.17e-04
D-Gluconic acid	0.9140	0.4800	1.3500	7.42e-05	3.17e-04
Pyruvic acid	0.8460	0.4240	1.2700	1.53e-04	5.98e-04
N-Methyl-DL-Alanine	-0.6680	-1.0400	-0.2960	6.27e-04	2.27e-03
L-α-Aminobutyric acid	-0.6520	-1.0300	-0.2780	8.66e-04	2.88e-03
Methionine	-0.6590	-1.0400	-0.2790	9.18e-04	2.88e-03
Ornithine	-0.6240	-0.9870	-0.2620	9.87e-04	2.90e-03
L-2-Aminobutyric acid	-0.6400	-1.0200	-0.2620	1.17e-03	3.20e-03
Serine	-0.6840	-1.0900	-0.2790	1.22e-03	3.20e-03
L-Norvaline	-0.6050	-0.9760	-0.2350	1.72e-03	4.27e-03
L-5-Oxoproline	-0.6390	-1.0300	-0.2430	1.94e-03	4.55e-03
Phenylalanine	-0.5960	-0.9680	-0.2240	2.10e-03	4.69e-03
Threonine	-0.6050	-0.9990	-0.2110	3.10e-03	6.62e-03
Palmitoleic acid	0.6800	0.2320	1.1300	3.45e-03	6.90e-03
Proline	-0.6440	-1.0700	-0.2160	3.72e-03	6.90e-03
Linoleic acid	0.6800	0.2270	1.1300	3.78e-03	6.90e-03
Palmitic acid	0.7090	0.2360	1.1800	3.82e-03	6.90e-03
Stearic acid	0.6510	0.2030	1.1000	4.99e-03	8.68e-03
Tyrosine	-0.4890	-0.8330	-0.1460	5.89e-03	9.89e-03
N-Acetyl-D-glucosamine	0.5570	0.0897	1.0200	2.02e-02	3.27e-02
4-Methylvaleric acid	-0.4420	-0.8280	-0.0556	2.56e-02	4.00e-02
Lysine	-0.3790	-0.7300	-0.0281	3.47e-02	5.26e-02
Deconaic acid	0.4680	0.0317	0.9040	3.59e-02	5.27e-02
Glycine	-0.3810	-0.7410	-0.0213	3.82e-02	5.44e-02
Alanine	-0.4010	-0.7900	-0.0120	4.35e-02	6.02e-02
Aspartic acid	0.3480	-0.0908	0.7860	1.18e-01	1.59e-01
L-Tryptophan	-0.3330	-0.7970	0.1320	1.58e-01	2.07e-01
Maltose	-0.3420	-0.8480	0.1640	1.82e-01	2.31e-01
Isobutylamine	-0.2340	-0.6520	0.1840	2.69e-01	3.33e-01
Glutamic acid	-0.2100	-0.6040	0.1850	2.93e-01	3.53e-01
L-Histidine	-0.1950	-0.5710	0.1800	3.03e-01	3.56e-01
Glutamine	-0.1860	-0.5820	0.2100	3.52e-01	4.03e-01
D-(+)-Xylose	-0.0871	-0.4640	0.2900	6.46e-01	7.23e-01
3-Indoleacetic acid	-0.0583	-0.4440	0.3270	7.64e-01	8.35e-01
D-Galactose	-0.0572	-0.4960	0.3820	7.96e-01	8.50e-01
Malonic acid	0.0444	-0.4340	0.5230	8.54e-01	8.73e-01
Glyoxylic acid	-0.0372	-0.4410	0.3670	8.55e-01	8.73e-01
3-Hydroxybutyric acid	0.0312	-0.4400	0.5030	8.95e-01	8.95e-01

Electronic Supplementary Material Table 3a: Impact of hypoglycaemic exposure using time below range level 1. Metabolomics data before and after the completion of the adrenaline infusions of the participants with type 1 diabetes before were assessed using linear mixed-effects model and TBR level 1 as fixed factor. Data are presented as a coefficient with confidence interval. Raw P-values are shown and the adjusted P-values using Benjamini-Hochberg.

Metabolites	Coefficient	95% Confidence interval		P	P_{BH}
		Lower	Upper		
L-Hydroxyproline	-12.70	-21.80	-3.50	0.01	0.47
DL-Pyroglutamic acid	-12.50	-21.70	-3.27	0.01	0.47
3-Hydroxyisovaleric acid	12.20	3.14	21.40	0.01	0.47
Octanoic acid	-11.60	-20.60	-2.56	0.01	0.48
N-Acetyl-D-glucosamine	10.80	2.10	19.50	0.02	0.48
Palmitic acid	-11.00	-20.00	-1.92	0.02	0.48
Linoleic acid	-10.40	-19.30	-1.42	0.02	0.48
L-Tryptophan	10.60	1.42	19.70	0.02	0.48
Stearic acid	-9.94	-18.90	-0.97	0.03	0.53
Methyl stearate	-9.29	-18.10	-0.44	0.04	0.53
Oleic acid	-9.43	-18.40	-0.43	0.04	0.53
Glutamic acid	9.37	0.35	18.40	0.04	0.53
Nonanoic acid	-9.30	-18.30	-0.33	0.04	0.53
Arachidonic acid	-8.67	-17.50	0.20	0.06	0.61
Methyl linoleate	-8.60	-17.40	0.23	0.06	0.61
Uric acid	-8.56	-17.50	0.42	0.06	0.62
Heptanoic acid	-8.28	-17.30	0.75	0.07	0.69
Aminomalonic acid	-7.82	-16.80	1.14	0.09	0.78
Acetoacetic acid	7.78	-1.42	17.00	0.10	0.83
α-Ketoglutaric acid	7.31	-1.64	16.30	0.11	0.88
Ribitol	-7.05	-16.00	1.91	0.12	0.92
2-Methylglutaric acid	-7.10	-16.20	1.98	0.13	0.92
L-Pipecolic acid	-6.83	-15.80	2.09	0.13	0.92
Tetradecanoic acid	-6.76	-15.70	2.21	0.14	0.92
Nonacosane	-6.75	-15.90	2.44	0.15	0.92
Dodecanoic acid	-6.53	-15.50	2.49	0.16	0.92
Methyl hexadecanoic acid	-6.26	-15.20	2.68	0.17	0.92
Decanoic acid	-6.29	-15.30	2.70	0.17	0.92
Hypoxanthine	6.35	-2.73	15.40	0.17	0.92
Pseudo uridine	-6.19	-15.10	2.69	0.17	0.92
Phosphoric acid 2,3-dihydroxypropyl ester	-6.08	-15.00	2.84	0.18	0.94
Trehalose	-5.71	-14.40	3.03	0.20	0.98
1-Monopalmitin	-5.72	-14.50	3.09	0.20	0.98
β-Tocopherol	-5.64	-14.50	3.21	0.21	0.98
L-5-Oxoproline	-5.59	-14.50	3.26	0.22	0.98
2-Palmitoylglycerol	-5.55	-14.40	3.31	0.22	0.98
Cholesterol	-5.55	-14.50	3.45	0.23	0.99
D-Galactose (1E)	5.49	-3.53	14.50	0.23	0.99
D-Glucose 6-phosphate	-5.22	-14.50	4.03	0.27	0.99
Phosphonoacetate	-4.91	-14.00	4.19	0.29	0.99
α-Tocopherol	-4.65	-13.60	4.29	0.31	0.99
Ketovaline	-4.59	-13.60	4.38	0.31	0.99
Glutamic acid-d5	4.35	-4.37	13.10	0.33	0.99
D-(+)-Trehalose	-4.52	-13.80	4.74	0.34	0.99
2-Methyl-6-tert-butylphenol	-4.32	-13.40	4.77	0.35	0.99
Aspartic acid	4.21	-4.80	13.20	0.36	0.99
2-Ketoisocaproic acid	4.40	-5.08	13.90	0.36	0.99
Palmitoleic acid	-4.08	-13.10	4.97	0.38	0.99
Fumaric acid	-4.05	-13.10	5.02	0.38	0.99
D-Glucarate	-3.88	-12.70	4.92	0.39	0.99
D-(-)-Rhamnose	-3.88	-12.80	5.03	0.39	0.99

Linolenic acid	-3.82	-12.70	5.02	0.40	0.99
Ethanolamine	-3.83	-12.70	5.06	0.40	0.99
2-Hydroxypyridine	-3.80	-12.90	5.27	0.41	0.99
Glyceryl-glycoside	-3.57	-12.30	5.17	0.42	0.99
2,3-Dihydroxybutanoic acid	-3.48	-12.30	5.37	0.44	0.99
Pyrophosphate	3.42	-5.42	12.30	0.45	0.99
3-Pyridinol	-3.41	-12.40	5.55	0.45	0.99
N-Acetylneuraminate	-3.19	-12.00	5.58	0.47	0.99
Heptadecanoate	-3.32	-12.50	5.86	0.48	0.99
Ribonic acid	-3.17	-11.90	5.60	0.48	0.99
Levoglucosan	-3.16	-12.00	5.66	0.48	0.99
4-amino-3-hydroxybutanoic acid	-3.03	-11.80	5.74	0.50	0.99
Glycine	-2.73	-11.50	6.04	0.54	0.99
Dihydrouracil	-2.73	-11.50	6.05	0.54	0.99
2,5-di-tert-butyl-1,4-benzoquinone	2.76	-6.24	11.80	0.55	0.99
Glyoxylic acid	-2.70	-11.60	6.21	0.55	0.99
Tetradecanol	-2.77	-11.90	6.39	0.55	0.99
Methylmalonic acid	-2.66	-11.50	6.20	0.56	0.99
n-Butylamine	-2.75	-11.90	6.42	0.56	0.99
Ornithine	-2.62	-11.30	6.11	0.56	0.99
Triethylene glycol	-2.62	-11.40	6.20	0.56	0.99
3-Hydroxybutyric acid	-2.71	-11.90	6.47	0.56	0.99
Methionine	2.57	-6.24	11.40	0.57	0.99
4,4-Dibromooctafluorobiphenyl	2.47	-6.21	11.20	0.58	0.99
Decenoyl-ACP	-2.52	-11.40	6.32	0.58	0.99
Parabanic acid	2.47	-6.42	11.40	0.59	0.99
Malic acid	-2.48	-11.50	6.56	0.59	0.99
D-(+)-Galacturonic acid	2.47	-6.60	11.50	0.59	0.99
D-Galactose (1Z)	-2.39	-11.20	6.42	0.59	0.99
Tryptophan	2.44	-6.57	11.40	0.59	0.99
1.6-Anhydroglucose	-2.31	-11.00	6.43	0.60	0.99
Succinic acid	-2.32	-11.20	6.53	0.61	0.99
p-Cresol	-2.39	-11.60	6.78	0.61	0.99
Ibuprofen	2.27	-6.54	11.10	0.61	0.99
3-Indoleacetic acid	-2.23	-11.10	6.59	0.62	0.99
Sorbitol	-2.26	-11.30	6.78	0.62	0.99
Glutamine	2.20	-6.70	11.10	0.63	0.99
Maltose	-2.21	-11.20	6.74	0.63	0.99
Oxalic acid	-2.21	-11.30	6.90	0.63	0.99
Pyruvic acid	2.14	-6.75	11.00	0.64	0.99
n-Propylamine	-2.12	-10.90	6.70	0.64	0.99
Diethanolamine	2.19	-7.01	11.40	0.64	0.99
D-(-)-Tagatofuranose	2.18	-6.99	11.40	0.64	0.99
D-(+)-Xylose	-2.11	-11.00	6.77	0.64	0.99
Proline	-2.09	-11.00	6.82	0.64	0.99
β-D-Glucopyranose	2.08	-6.90	11.10	0.65	0.99
3-Indolepropionic acid	-2.02	-10.80	6.75	0.65	0.99
β-Alanine	-2.00	-10.80	6.80	0.66	0.99
L-Histidine	1.94	-6.92	10.80	0.67	0.99
2-hydroxypropanoic acid	1.94	-6.97	10.80	0.67	0.99
3-Hydroxyicosatetraenoyl-CoA	-2.00	-11.30	7.25	0.67	0.99
Isoleucine	1.87	-6.88	10.60	0.68	0.99
Valine	-1.84	-10.50	6.84	0.68	0.99
Glyceric acid	-1.87	-10.70	6.95	0.68	0.99
Melibiose	1.75	-7.04	10.50	0.70	0.99
Valine-d8	1.72	-7.03	10.50	0.70	0.99
Hydroquinone	-1.67	-10.30	6.98	0.71	0.99
D-Fucitol	-1.69	-10.50	7.11	0.71	0.99
Benzoic acid	1.76	-7.45	11.00	0.71	0.99
Succinic acid-d4	1.64	-7.03	10.30	0.71	0.99

Serine	-1.63	-10.50	7.25	0.72	0.99
d-Glucose	-1.63	-10.60	7.34	0.72	0.99
L-Norleucine	1.57	-7.19	10.30	0.72	0.99
Alanine	-1.55	-10.40	7.31	0.73	0.99
Taurine	-1.58	-10.80	7.67	0.74	0.99
L-Threonic acid	-1.45	-10.20	7.28	0.74	0.99
L-Arginine	1.43	-7.18	10.00	0.74	0.99
Putrescine	-1.41	-10.30	7.45	0.75	0.99
Malonic acid	-1.43	-10.60	7.75	0.76	0.99
Docosahexaenoic acid	-1.36	-10.20	7.48	0.76	0.99
1,3-Propanediol	1.36	-7.55	10.30	0.76	0.99
2-Keto-3-methylpentanoic acid	-1.40	-10.60	7.84	0.77	0.99
1-Dodecanol	-1.34	-10.40	7.73	0.77	0.99
Eicosapentaenoic acid	-1.12	-9.91	7.67	0.80	0.99
Citric acid	1.12	-7.74	9.97	0.80	0.99
Leucine	1.08	-7.69	9.85	0.81	0.99
Myo-inositol	-1.08	-9.86	7.70	0.81	0.99
Glycolic acid	-1.04	-9.77	7.69	0.82	0.99
Heptadecanoic acid-d33	0.99	-7.73	9.70	0.82	0.99
Threonine	-1.00	-9.87	7.87	0.82	0.99
Phenoxyacetic acid	-0.97	-9.79	7.85	0.83	0.99
D-(-)-Lyxofuranose	0.97	-7.99	9.93	0.83	0.99
Tyrosine	-0.92	-9.65	7.81	0.84	0.99
Lysine	0.91	-7.86	9.67	0.84	0.99
Acetylsalicylic acid	0.91	-7.97	9.80	0.84	0.99
Cysteine	0.90	-8.16	9.95	0.85	0.99
D-Gluconic acid	0.84	-8.04	9.71	0.85	0.99
Phosphoric acid	0.71	-8.25	9.66	0.88	0.99
Butyl hexadecanoic acid	0.70	-8.52	9.91	0.88	0.99
α-D-(-)-Tagatopyran	-0.70	-10.10	8.67	0.88	0.99
N-Methyl-DL-Alanine	-0.66	-9.47	8.16	0.88	0.99
Isobutylamine	-0.66	-9.69	8.37	0.89	0.99
L-2-Aminobutyric acid	-0.63	-9.45	8.20	0.89	0.99
L-(-)-Sorbose	0.59	-8.17	9.36	0.89	0.99
L-Norvaline	-0.53	-9.30	8.24	0.91	0.99
L-α-Aminobutyric acid	-0.53	-9.34	8.28	0.91	0.99
Diethylene glycol	0.53	-8.42	9.48	0.91	0.99
1,5-Anhydrohexitol	-0.49	-9.36	8.38	0.91	0.99
Ketoisoleucine	-0.50	-9.88	8.88	0.92	0.99
Phosphoric acid, monomethyl ester	0.46	-8.40	9.31	0.92	0.99
D-Arabitol	-0.32	-9.24	8.60	0.94	0.99
L-(-)-Arabitol	0.29	-8.39	8.97	0.95	0.99
O-Phosphoethanolamine	-0.29	-9.17	8.60	0.95	0.99
Phenylalanine	-0.26	-9.10	8.57	0.95	0.99
Urea	0.26	-8.58	9.09	0.96	0.99
4-Methylvaleric acid	-0.24	-9.08	8.60	0.96	0.99
Glycerol	-0.16	-9.22	8.90	0.97	0.99
2-Hydroxybutyric acid	-0.14	-8.95	8.66	0.97	0.99
2-Desoxy-D-glycero-pentos-3-ulose	0.077	-8.76	8.92	0.99	0.99
Quininic acid	-0.064	-8.80	8.68	0.99	0.99
Meso-erythritol	0.052	-8.76	8.87	0.99	0.99

Electronic Supplementary Material Table 3b: Impact of hypoglycaemic exposure using time below range level 2. Metabolomics data before and after the completion of the adrenaline infusions of the participants with type 1 diabetes before were assessed using linear mixed-effects model and TBR level 2 as fixed factor. Data are presented as a coefficient with confidence interval. Raw P-values are shown and the adjusted P-values using Benjamini-Hochberg.

Metabolites	Coefficient	95% Confidence interval		P	P_{BH}
		Lower	Upper		
N-Acetyl-D-glucosamine	37.00	10.80	63.30	0.01	0.52
L-Tryptophan	37.90	10.40	65.50	0.01	0.52
Palmitic acid	-34.90	-62.20	-7.67	0.01	0.52
Oleic acid	-32.80	-59.90	-5.71	0.02	0.52
L-Hydroxyproline	-33.60	-61.40	-5.82	0.02	0.52
Linoleic acid	-31.80	-58.80	-4.72	0.02	0.52
Stearic acid	-31.10	-58.20	-4.02	0.02	0.52
Methyl stearate	-29.90	-56.60	-3.18	0.03	0.52
3-Hydroxyisovaleric acid	30.90	3.18	58.50	0.03	0.52
Arachidonic acid	-28.30	-55.00	-1.51	0.04	0.57
Uric acid	-28.60	-55.70	-1.51	0.04	0.57
Tetradecanoic acid	-27.80	-54.70	-0.78	0.04	0.59
2-Ketoisocaproic acid	28.70	0.25	57.20	0.05	0.60
Octanoic acid	-26.90	-54.30	0.45	0.05	0.62
Heptadecanoate	-26.40	-54.00	1.26	0.06	0.66
Methyl linoleate	-24.50	-51.20	2.14	0.07	0.68
Palmitoleic acid	-25.00	-52.20	2.23	0.07	0.68
Acetoacetic acid	25.00	-2.78	52.90	0.08	0.69
α-Ketoglutaric acid	23.60	-3.25	50.40	0.08	0.69
Nonanoic acid	-23.80	-50.90	3.34	0.09	0.69
Dodecanoic acid	-21.90	-49.10	5.34	0.12	0.88
DL-Pyroglutamic acid	-22.40	-50.50	5.80	0.12	0.88
n-Butylamine	-21.00	-48.60	6.68	0.14	0.93
Heptanoic acid	-20.70	-48.00	6.64	0.14	0.93
Glutamic acid	20.10	-7.37	47.50	0.15	0.97
2,5-di-tert-Butyl-1,4-benzoquinone	19.60	-7.53	46.70	0.16	0.97
Decanoic acid	-19.00	-46.10	8.09	0.17	0.999
Methyl hexadecanoic acid	-18.20	-45.20	8.85	0.19	0.999
Pyruvic acid	17.90	-9.00	44.80	0.19	0.999
Parabanic acid	17.90	-9.03	44.80	0.19	0.999
3-Hydroxybutyric acid	-17.20	-44.80	10.50	0.22	0.999
Phosphoric acid	15.90	-11.20	43.00	0.25	0.999
Pseudo uridine	-15.20	-42.10	11.60	0.27	0.999
D-(+)-Trehalose	-15.80	-43.80	12.20	0.27	0.999
Trehalose	-14.90	-41.50	11.60	0.27	0.999
2-Methylglutaric acid	-15.40	-42.80	12.10	0.27	0.999
Malonic acid	-15.30	-43.00	12.40	0.28	0.999
D-Galactose (1E)	15.00	-12.30	42.30	0.28	0.999
1-Monopalmitin	-14.70	-41.40	12.10	0.28	0.999
Pyrophosphate	14.50	-12.20	41.20	0.29	0.999
1,3-Propanediol	13.90	-13.00	40.80	0.31	0.999
2-Palmitoylglycerol	-13.60	-40.50	13.20	0.32	0.999
2-Hydroxypropanoic acid	13.40	-13.50	40.30	0.33	0.999
Ribitol	-13.10	-40.30	14.10	0.34	0.999
D-(+)-Galacturonic acid	13.10	-14.30	40.50	0.35	0.999
Glutamic acid-d5	12.60	-13.80	39.00	0.35	0.999
Aminomalonic acid	-12.80	-40.10	14.50	0.36	0.999
Melibiose	12.10	-14.50	38.60	0.37	0.999
Isoleucine	11.60	-14.80	38.10	0.39	0.999
β-D-Glucopyranose	11.90	-15.30	39.10	0.39	0.999
L-Norleucine	11.30	-15.10	37.80	0.40	0.999
Leucine	10.80	-15.70	37.30	0.42	0.999

Phosphoric acid 2,3-dihydroxypropyl ester	-10.80	-37.80	16.30	0.43	0.999
Methionine	10.60	-16.10	37.30	0.44	0.999
D-Gluconic acid	10.60	-16.30	37.50	0.44	0.999
Isobutylamine	10.20	-17.00	37.50	0.46	0.999
β-Tocopherol	-8.90	-35.80	18.00	0.52	0.999
Diethanolamine	9.09	-18.70	36.90	0.52	0.999
2-Methyl-6-tert-butylphenol	-8.93	-36.40	18.60	0.52	0.999
Glyceryl-glycoside	-8.45	-34.90	18.00	0.53	0.999
Valine-d8	8.32	-18.20	34.80	0.54	0.999
Tetradecanol	-8.51	-36.20	19.20	0.55	0.999
D-(-)-Rhamnose	-8.01	-34.90	18.90	0.56	0.999
Succinic acid	-7.94	-34.70	18.80	0.56	0.999
Hypoxanthine	7.75	-19.80	35.30	0.58	0.999
D-Glucarate	-7.25	-33.90	19.40	0.59	0.999
Citric acid	7.23	-19.60	34.00	0.60	0.999
Linolenic acid	-7.22	-34.00	19.50	0.60	0.999
Phosphoric acid, monomethyl ester	7.23	-19.60	34.00	0.60	0.999
Levoglucosan	-7.19	-33.90	19.50	0.60	0.999
Triethylene glycol	-7.16	-33.80	19.50	0.60	0.999
Succinic acid-d4	6.79	-19.50	33.10	0.61	0.999
4,4-Dibromooctafluorobiphenyl	6.78	-19.60	33.10	0.61	0.999
L-α-Aminobutyric acid	6.46	-20.20	33.10	0.63	0.999
4-Amino-3-hydroxybutanoic acid	-6.31	-32.90	20.30	0.64	0.999
L-Norvaline	6.29	-20.20	32.80	0.64	0.999
N-Acetylneuraminate	-6.28	-32.80	20.30	0.64	0.999
Meso-erythritol	6.26	-20.40	32.90	0.64	0.999
N-Methyl-DL-alanine	6.24	-20.40	32.90	0.65	0.999
Benzoic acid	6.48	-21.40	34.30	0.65	0.999
D-Glucose	6.22	-20.90	33.30	0.65	0.999
Butyl hexadecanoic acid	-6.36	-34.30	21.50	0.65	0.999
D-Glucose 6-phosphate	6.37	-21.60	34.40	0.66	0.999
L-Histidine	6.05	-20.80	32.90	0.66	0.999
Putrescine	5.86	-21.00	32.70	0.67	0.999
L-2-Aminobutyric acid	5.77	-20.90	32.50	0.67	0.999
1-Dodecanol	-5.77	-33.10	21.60	0.68	0.999
L-5-Oxoproline	-5.67	-32.60	21.20	0.68	0.999
Urea	-5.24	-32.00	21.50	0.70	0.999
L-Arginine	4.91	-21.10	31.00	0.71	0.999
1.6-Anhydroglucose	-4.84	-31.30	21.60	0.72	0.999
Phenylalanine	4.78	-22.00	31.50	0.73	0.999
Taurine	-4.98	-33.00	23.00	0.73	0.999
Alanine	4.72	-22.10	31.50	0.73	0.999
Ornithine	-4.61	-31.10	21.80	0.73	0.999
α-D-(-)-Tagatopyranose	4.79	-23.50	33.10	0.74	0.999
p-Cresol	-4.46	-32.20	23.30	0.75	0.999
Glutamine	4.29	-22.70	31.30	0.75	0.999
L-(-)-Arabitol	4.15	-21.90	30.20	0.75	0.999
2,3-Dihydroxybutanoic acid	-4.25	-31.10	22.60	0.76	0.999
Aspartic acid	3.95	-23.30	31.20	0.78	0.999
Phenoxyacetic acid	3.86	-22.90	30.60	0.78	0.999
Oxalic acid	3.96	-23.60	31.50	0.78	0.999
Ribonic acid	-3.82	-30.40	22.70	0.78	0.999
d-Galactose (1Z)	-3.82	-30.40	22.80	0.78	0.999
L-Pipecolic acid	-3.87	-30.90	23.20	0.78	0.999
Serine	3.80	-23.10	30.70	0.78	0.999
D-Arabitol	3.72	-23.30	30.70	0.79	0.999
3-Indolepropionic acid	-3.58	-30.10	23.00	0.79	0.999
Malic acid	-3.68	-31.00	23.70	0.79	0.999
D-(-)-Tagatofuranose	3.62	-24.10	31.40	0.80	0.999

Threonine	3.47	-23.40	30.30	0.80	0.999
Ketovaline	-3.42	-30.70	23.80	0.81	0.999
Tryptophan	3.24	-24.10	30.60	0.82	0.999
D-(-)-Lyxofuranose	3.21	-23.90	30.30	0.82	0.999
Proline	3.18	-23.80	30.20	0.82	0.999
Valine	-3.09	-29.40	23.20	0.82	0.999
Cholesterol	-3.18	-30.50	24.20	0.82	0.999
Cysteine	-3.02	-30.00	24.00	0.83	0.999
Glyceric acid	-2.93	-29.60	23.70	0.83	0.999
Glycolic acid	2.90	-23.50	29.30	0.83	0.999
α-Tocopherol	-2.97	-30.10	24.20	0.83	0.999
2-Keto-3-methylpentanoic acid	3.04	-25.00	31.00	0.83	0.999
4-Methylvaleric acid	2.86	-23.90	29.60	0.83	0.999
L-(-)-Sorbose	2.49	-24.00	29.00	0.85	0.999
Ibuprofen	2.36	-24.30	29.00	0.86	0.999
Fumaric acid	-2.20	-29.70	25.30	0.88	0.999
Sorbitol	-2.05	-29.50	25.30	0.88	0.999
Tyrosine	-1.92	-28.40	24.50	0.89	0.999
Dihydrouracil	-1.84	-28.40	24.70	0.89	0.999
β-Alanine	-1.82	-28.50	24.90	0.89	0.999
Quininic acid	1.80	-24.70	28.30	0.89	0.999
2-Hydroxypyridine	1.77	-25.70	29.20	0.90	0.999
n-Propylamine	1.67	-25.00	28.30	0.90	0.999
Nonacosane	-1.67	-29.60	26.20	0.91	0.999
Glycine	-1.58	-28.20	25.00	0.91	0.999
D-Fucitol	1.53	-25.10	28.20	0.91	0.999
Docosahexaenoic acid	-1.52	-28.20	25.20	0.91	0.999
Lysine	1.45	-25.10	28.00	0.92	0.999
Ethanolamine	1.25	-25.60	28.10	0.93	0.999
Glycerol	1.16	-26.20	28.60	0.93	0.999
Phosphonoacetate	-1.14	-28.70	26.40	0.94	0.999
2-Desoxy-D-glycero-pentos-3-ulose	0.99	-25.80	27.80	0.94	0.999
2-Hydroxybutyric acid	-0.93	-27.60	25.70	0.95	0.999
Acetylsalicylic acid	-0.91	-27.80	25.90	0.95	0.999
D-(+)-Xylose	0.88	-26.00	27.80	0.95	0.999
Diethylene glycol	-0.84	-27.90	26.20	0.95	0.999
Ketoisoleucine	0.81	-27.60	29.20	0.96	0.999
L-Threonic acid	0.73	-25.80	27.20	0.96	0.999
(2E)-Decenoyl-ACP	0.67	-26.10	27.40	0.96	0.999
3-Indoleacetic acid	-0.65	-27.40	26.10	0.96	0.999
O-Phosphoethanolamine	0.63	-26.20	27.50	0.96	0.999
3-Pyridinol	-0.56	-27.70	26.60	0.97	0.999
Heptadecanoic acid-d33	-0.48	-26.90	25.90	0.97	0.999
Glyoxylic acid	-0.39	-27.30	26.60	0.98	0.999
Methylmalonic acid	-0.34	-27.10	26.50	0.98	0.999
Hydroquinone	-0.31	-26.50	25.90	0.98	0.999
Eicosapentaenoic acid	-0.26	-26.80	26.30	0.99	0.999
1,5-Anhydrohexitol	-0.23	-27.10	26.60	0.99	0.999
Myo-inositol	0.14	-26.50	26.80	0.99	0.999
Maltose	0.04	-27.00	27.10	1.00	0.999
3-Hydroxyicosatetraenoyl-CoA	-0.02	-28.00	28.00	1.00	0.999

Electronic Supplementary Material Table 3c: Impact of hypoglycaemic exposure using hypoglycaemic events per week level 1. Metabolomics data before and after the completion of the adrenaline infusions of the participants with type 1 diabetes before were assessed using linear mixed-effects model and HEPW level 1 as fixed factor. Data are presented as a coefficient with confidence interval. Raw P-values are shown and the adjusted P-values using Benjamini-Hochberg.

Metabolites	Coefficient	95% Confidence interval		P	P_{BH}
		Lower	Upper		
DL-Pyroglutamic acid	-0.23	-0.33	-0.13	< 0.001	0.004
Octanoic acid	-0.16	-0.26	-0.058	0.002	0.20
Nonacosane	-0.16	-0.26	-0.050	0.004	0.20
Phosphonoacetate	-0.15	-0.25	-0.046	0.005	0.20
L-Pipecolic acid	-0.14	-0.24	-0.035	0.01	0.23
2-Methylglutaric acid	-0.14	-0.24	-0.035	0.01	0.23
D-Glucose 6-phosphate	-0.14	-0.25	-0.034	0.01	0.23
Ketovaline	-0.13	-0.23	-0.026	0.01	0.28
Cholesterol	-0.13	-0.23	-0.024	0.02	0.28
Nonanoic acid	-0.12	-0.22	-0.018	0.02	0.31
Aminomalonic acid	-0.12	-0.22	-0.018	0.02	0.31
2-Hydroxypyridine	-0.12	-0.22	-0.014	0.03	0.31
Fumaric acid	-0.12	-0.22	-0.014	0.03	0.31
Phosphoric acid 2,3-dihydroxypropyl ester	-0.11	-0.22	-0.012	0.03	0.31
3-Hydroxyisovaleric acid	0.12	0.01	0.22	0.03	0.31
Ribitol	-0.11	-0.21	-0.009	0.03	0.31
Glutamic acid	0.11	0.01	0.22	0.03	0.31
Methyl linoleate	-0.11	-0.21	-0.008	0.03	0.31
L-Tryptophan	0.11	0.01	0.22	0.04	0.31
β-Tocopherol	-0.11	-0.21	-0.005	0.04	0.31
Linoleic acid	-0.11	-0.21	-0.004	0.04	0.31
Ethanolamine	-0.11	-0.21	-0.003	0.04	0.31
α-Tocopherol	-0.11	-0.21	-0.003	0.04	0.31
2-Methyl-6-tert-butylphenol	-0.10	-0.21	0.001	0.05	0.35
D-(-)-Rhamnose	-0.10	-0.20	0.005	0.06	0.38
Heptanoic acid	-0.10	-0.20	0.005	0.06	0.38
L-Hydroxyproline	-0.10	-0.21	0.006	0.07	0.38
Stearic acid	-0.10	-0.20	0.007	0.07	0.38
Maltose	-0.095	-0.20	0.008	0.07	0.39
Methyl hexadecanoic acid	-0.092	-0.20	0.010	0.08	0.40
L-5-Oxoproline	-0.091	-0.19	0.010	0.08	0.40
Pseudo uridine	-0.091	-0.19	0.011	0.08	0.40
N-Acetyl-D-glucosamine	0.089	-0.01	0.19	0.08	0.41
Methyl stearate	-0.089	-0.19	0.013	0.09	0.41
3-Hydroxyicosatetraenoyl-CoA	-0.092	-0.20	0.014	0.09	0.41
Glyoxylic acid	-0.086	-0.19	0.016	0.10	0.44
Decanoic acid	-0.087	-0.19	0.017	0.10	0.44
Palmitic acid	-0.086	-0.19	0.018	0.11	0.45
Proline	-0.080	-0.18	0.022	0.12	0.51
1-Monopalmitin	-0.077	-0.18	0.023	0.13	0.52
Methylmalonic acid	-0.078	-0.18	0.024	0.13	0.52
d-Glucose	-0.078	-0.18	0.025	0.14	0.52
α-D-(-)-Tagatopyranose	-0.082	-0.19	0.027	0.14	0.52
Isobutylamine	-0.078	-0.18	0.026	0.14	0.52
Decenoyl-ACP	-0.074	-0.18	0.027	0.15	0.54
Putrescine	-0.072	-0.17	0.029	0.16	0.54
Oxalic acid	-0.074	-0.18	0.031	0.16	0.54
n-Propylamine	-0.071	-0.17	0.030	0.17	0.54
Sorbitol	-0.073	-0.18	0.031	0.17	0.54
Uric acid	-0.072	-0.18	0.032	0.17	0.54
D-Fucitol	-0.069	-0.17	0.031	0.17	0.54

3-Pyridinol	-0.071	-0.17	0.032	0.18	0.54
D-Galactose (1Z)	-0.069	-0.17	0.032	0.18	0.54
D-Gluconic acid	-0.068	-0.17	0.034	0.19	0.57
p-Cresol	-0.070	-0.18	0.036	0.19	0.57
Glycolic acid	-0.065	-0.16	0.035	0.20	0.57
Alanine	-0.066	-0.17	0.036	0.20	0.57
2-Palmitoylglycerol	-0.066	-0.17	0.036	0.20	0.57
D-(+)-Xylose	-0.065	-0.17	0.037	0.21	0.57
Phosphoric acid	-0.065	-0.17	0.038	0.21	0.57
Oleic acid	-0.066	-0.17	0.039	0.22	0.57
N-Acetylneuraminate	-0.063	-0.16	0.037	0.22	0.57
Aspartic acid	0.064	-0.040	0.17	0.22	0.57
Tryptophan	0.064	-0.040	0.17	0.23	0.57
Dodecanoic acid	-0.064	-0.17	0.041	0.23	0.57
D-(+)-Trehalose	-0.065	-0.17	0.042	0.23	0.57
2,3-Dihydroxybutanoic acid	-0.061	-0.16	0.040	0.24	0.57
3-Indoleacetic acid	-0.060	-0.16	0.041	0.25	0.58
Eicosapentaenoic acid	-0.058	-0.16	0.042	0.25	0.58
Butyl hexadecanoic acid	0.061	-0.045	0.17	0.26	0.58
Parabanic acid	-0.059	-0.16	0.044	0.26	0.58
Trehalose	-0.057	-0.16	0.043	0.26	0.58
D-Galactose (1E)	0.059	-0.045	0.16	0.26	0.58
3-Indolepropionic acid	-0.057	-0.16	0.043	0.26	0.58
Serine	-0.057	-0.16	0.044	0.27	0.58
Linolenic acid	-0.057	-0.16	0.045	0.27	0.58
Dihydrouracil	-0.055	-0.16	0.046	0.29	0.58
Ribonic acid	-0.054	-0.15	0.046	0.29	0.58
D-Glucarate	-0.054	-0.16	0.046	0.29	0.58
Arachidonic acid	-0.055	-0.16	0.047	0.29	0.58
Tetradecanol	-0.057	-0.16	0.049	0.29	0.58
Glutamic acid-d5	0.053	-0.047	0.15	0.30	0.58
Glycine	-0.054	-0.15	0.047	0.30	0.58
Malic acid	-0.055	-0.16	0.049	0.30	0.58
D-Arabitol	-0.051	-0.15	0.051	0.33	0.61
Ibuprofen	0.050	-0.051	0.15	0.33	0.61
N-Methyl-DL-alanine	-0.050	-0.15	0.051	0.33	0.61
Phenoxyacetic acid	-0.049	-0.15	0.052	0.34	0.61
Levoglucosan	-0.049	-0.15	0.052	0.34	0.61
L-2-Aminobutyric acid	-0.049	-0.15	0.053	0.35	0.61
Myo-inositol	-0.048	-0.15	0.053	0.35	0.61
Hydroquinone	-0.047	-0.15	0.052	0.35	0.61
L-α-Aminobutyric acid	-0.048	-0.15	0.053	0.35	0.61
Threonine	-0.048	-0.15	0.054	0.36	0.61
Glyceryl-glycoside	-0.047	-0.15	0.053	0.36	0.61
Hypoxanthine	0.049	-0.056	0.15	0.36	0.61
Glutamine	0.046	-0.055	0.15	0.37	0.61
Palmitoleic acid	0.048	-0.057	0.15	0.37	0.61
2-Ketoisocaproic acid	-0.050	-0.16	0.061	0.37	0.61
meso-Erythritol	-0.045	-0.15	0.055	0.38	0.61
Acetoacetic acid	0.047	-0.060	0.16	0.38	0.61
L-Histidine	0.044	-0.057	0.15	0.39	0.61
L-Norvaline	-0.044	-0.14	0.057	0.39	0.61
D-(-)-Tagatofuranose	-0.046	-0.15	0.060	0.40	0.61
Lysine	0.043	-0.057	0.14	0.40	0.61
Triethylene glycol	-0.044	-0.15	0.058	0.40	0.61
Glyceric acid	-0.043	-0.14	0.058	0.40	0.61
1,6-Anhydroglucose	-0.039	-0.14	0.061	0.45	0.66
β-Alanine	-0.039	-0.14	0.062	0.45	0.66
4-Amino-3-hydroxybutanoic acid	-0.039	-0.14	0.062	0.45	0.66
Taurine	0.040	-0.067	0.15	0.46	0.67

Cysteine	0.041	-0.069	0.15	0.47	0.67
Pyruvic acid	-0.037	-0.14	0.065	0.47	0.67
Heptadecanoic acid-d33	0.036	-0.063	0.14	0.47	0.67
1,3-Propanediol	-0.037	-0.14	0.065	0.48	0.67
α-Ketoglutaric acid	0.038	-0.068	0.14	0.48	0.67
L-Threonic acid	-0.035	-0.14	0.06	0.49	0.67
Malonic acid	0.038	-0.069	0.14	0.49	0.67
Phosphoric acid, monomethyl ester	-0.035	-0.14	0.067	0.50	0.68
2-Keto-3-methylpentanoic acid	-0.035	-0.14	0.073	0.53	0.71
Ketoisoleucine	0.035	-0.075	0.14	0.53	0.71
4,4-Dibromooctafluorobiphenyl	0.029	-0.069	0.13	0.56	0.74
2-Hydroxypropanoic acid	-0.030	-0.13	0.073	0.57	0.75
Docosahexaenoic acid	-0.028	-0.13	0.074	0.59	0.77
Ornithine	-0.027	-0.13	0.072	0.59	0.77
D-(-)-Lyxofuranose	0.027	-0.076	0.13	0.60	0.77
Heptadecanoate	0.028	-0.079	0.14	0.61	0.77
1-Dodecanol	-0.027	-0.13	0.078	0.61	0.77
Benzoic acid	-0.027	-0.13	0.080	0.62	0.78
L-(-)-Sorbose	-0.023	-0.12	0.077	0.65	0.81
4-Methylvaleric acid	-0.023	-0.12	0.078	0.66	0.81
2,5-di-tert-Butyl-1,4-benzoquinone	-0.021	-0.13	0.082	0.69	0.83
Pyrophosphate	0.021	-0.081	0.12	0.69	0.83
Leucine	-0.020	-0.12	0.081	0.70	0.83
Tetradecanoic acid	-0.020	-0.13	0.084	0.70	0.83
n-Butylamine	0.021	-0.086	0.13	0.70	0.83
Succinic acid	-0.019	-0.12	0.082	0.71	0.84
L-(-)-Arabitol	-0.016	-0.12	0.083	0.75	0.87
Citric acid	-0.017	-0.12	0.085	0.75	0.87
D-(+)-Galacturonic acid	-0.017	-0.12	0.088	0.76	0.87
β-D-Glucopyranose	-0.016	-0.12	0.088	0.76	0.88
Quininic acid	-0.015	-0.11	0.085	0.77	0.88
Diethanolamine	-0.015	-0.12	0.091	0.78	0.88
Diethylene glycol	0.015	-0.088	0.12	0.78	0.88
Methionine	0.013	-0.088	0.11	0.80	0.89
1.5-Anhydrohexitol	-0.013	-0.12	0.089	0.80	0.89
2-Desoxy-D-glycero-pentos-3-ulose	-0.012	-0.11	0.089	0.81	0.89
Urea	0.012	-0.089	0.11	0.81	0.89
Valine	-0.011	-0.11	0.089	0.83	0.90
Tyrosine	0.011	-0.089	0.11	0.83	0.90
L-Norleucine	-0.010	-0.11	0.091	0.85	0.91
Acetylsalicylic acid	-0.010	-0.11	0.093	0.85	0.91
Phenylalanine	-0.009	-0.11	0.093	0.87	0.91
Succinic acid-d4	0.009	-0.090	0.11	0.87	0.91
Glycerol	-0.008	-0.11	0.10	0.88	0.91
3-Hydroxybutyric acid	0.008	-0.10	0.12	0.88	0.91
Melibiose	-0.004	-0.11	0.10	0.94	0.97
O-Phosphoethanolamine	0.003	-0.10	0.11	0.96	0.98
Isoleucine	-0.002	-0.10	0.10	0.97	0.98
Valine-d8	-0.001	-0.10	0.10	0.98	0.995
L-Arginine	-0.001	-0.10	0.10	0.99	0.996
2-Hydroxybutyric acid	0.0002	-0.10	0.10	1.00	0.997

Electronic Supplementary Material Table 3d: Impact of hypoglycemic exposure using hypoglycemic events per week level 2. Metabolomics data before and after the completion of the adrenaline infusions of the participants with type 1 diabetes before were assessed using linear mixed-effects model and hypoglycemic events per week level 2 as fixed factor. Data are presented as a coefficient with confidence interval. Raw P-values are shown and the adjusted P-values using Benjamini-Hochberg.

Metabolites	Coefficient	95% Confidence interval		P	P_{BH}
		Lower	Upper		
n-Butylamine	-0.22	-0.38	-0.061	0.01	0.35
Palmitic acid	-0.22	-0.38	-0.059	0.01	0.35
Methyl stearate	-0.21	-0.36	-0.046	0.01	0.35
N-Acetyl-D-glucosamine	0.20	0.041	0.36	0.01	0.35
3-Hydroxyisovaleric acid	0.20	0.039	0.36	0.02	0.35
L-Tryptophan	0.20	0.037	0.36	0.02	0.35
Linoleic acid	-0.20	-0.36	-0.036	0.02	0.35
Oleic acid	-0.20	-0.36	-0.035	0.02	0.35
Stearic acid	-0.19	-0.35	-0.029	0.02	0.36
Octanoic acid	-0.19	-0.35	-0.027	0.02	0.36
Methyl linoleate	-0.18	-0.34	-0.023	0.02	0.37
Methyl hexadecanoic acid	-0.18	-0.34	-0.020	0.03	0.37
DL-Pyroglutamic acid	-0.17	-0.33	-0.006	0.04	0.50
Heptanoic acid	-0.17	-0.33	-0.005	0.04	0.50
Tetradecanoic acid	-0.16	-0.32	-0.001	0.05	0.53
Nonanoic acid	-0.16	-0.32	0.002	0.05	0.53
Uric acid	-0.16	-0.32	0.004	0.06	0.53
Glutamic acid	0.15	-0.011	0.31	0.07	0.59
L-Hydroxyproline	-0.15	-0.32	0.012	0.07	0.59
Taurine	0.15	-0.02	0.31	0.08	0.62
Arachidonic acid	-0.14	-0.30	0.017	0.08	0.62
α-Ketoglutaric acid	0.14	-0.025	0.30	0.10	0.69
Palmitoleic acid	-0.14	-0.30	0.025	0.10	0.69
Pyruvic acid	0.13	-0.031	0.29	0.11	0.77
Fumaric acid	-0.13	-0.29	0.037	0.13	0.84
Decanoic acid	-0.12	-0.28	0.043	0.15	0.94
Dodecanoic acid	-0.12	-0.28	0.045	0.16	0.95
Aminomalonic acid	-0.11	-0.27	0.049	0.17	0.99
2-Methyl-6-tert-butylphenol	-0.11	-0.27	0.051	0.18	0.99
2-Methylglutaric acid	-0.11	-0.27	0.053	0.19	0.99
D-(+)-Trehalose	-0.11	-0.27	0.054	0.19	0.99
Ribitol	-0.10	-0.26	0.059	0.22	0.99
2-Ketoisocaproic acid	0.10	-0.061	0.27	0.22	0.99
β-Tocopherol	-0.10	-0.26	0.063	0.23	0.99
Heptadecanoate	-0.10	-0.26	0.067	0.25	0.99
1-Monopalmitin	-0.093	-0.25	0.066	0.25	0.99
Acetylsalicylic acid	-0.092	-0.25	0.068	0.26	0.99
2-Palmitoylglycerol	-0.092	-0.25	0.068	0.26	0.99
Phosphoric acid 2,3-dihydroxypropyl ester	-0.090	-0.25	0.070	0.27	0.99
Glutamic acid-d5	0.089	-0.069	0.25	0.27	0.99
Diethylene glycol	-0.090	-0.25	0.070	0.27	0.99
Pseudo uridine	-0.089	-0.25	0.070	0.27	0.99
Pyrophosphate	0.087	-0.073	0.25	0.29	0.99
3-Hydroxybutyric acid	-0.086	-0.25	0.076	0.30	0.99
Trehalose	-0.084	-0.24	0.075	0.30	0.99
Phosphonoacetate	-0.083	-0.25	0.08	0.31	0.99
Ketoisoleucine	0.082	-0.083	0.25	0.33	0.99
L-5-Oxoproline	-0.079	-0.24	0.081	0.33	0.99
Ketovaline	-0.078	-0.24	0.083	0.34	0.99
Cholesterol	-0.077	-0.24	0.084	0.35	0.99
2-Hydroxypyridine	-0.075	-0.24	0.086	0.36	0.99

2,5-di-tert-Butyl-1,4-benzoquinone	0.074	-0.087	0.23	0.37	0.99
Malonic acid	-0.073	-0.24	0.089	0.38	0.99
Cysteine	-0.075	-0.24	0.091	0.38	0.99
L-Pipecolic acid	-0.071	-0.23	0.090	0.39	0.99
Ibuprofen	0.069	-0.09	0.23	0.39	0.99
Glyoxylic acid	-0.070	-0.23	0.090	0.39	0.99
Tetradecanol	-0.070	-0.23	0.092	0.40	0.99
α-Tocopherol	-0.069	-0.23	0.092	0.40	0.99
Linolenic acid	-0.065	-0.23	0.094	0.42	0.99
Methylmalonic acid	-0.065	-0.23	0.094	0.42	0.99
D-Galactose (1E)	0.065	-0.10	0.23	0.43	0.99
Aspartic acid	0.065	-0.10	0.23	0.43	0.99
Levoglucosan	-0.063	-0.22	0.10	0.44	0.99
p-Cresol	-0.063	-0.23	0.10	0.44	0.99
2,3-Dihydroxybutanoic acid	-0.059	-0.22	0.10	0.47	0.99
Melibiose	0.057	-0.10	0.22	0.48	0.99
1-Dodecanol	-0.057	-0.22	0.10	0.49	0.99
β-Alanine	-0.054	-0.21	0.11	0.50	0.99
Diethanolamine	0.056	-0.11	0.22	0.50	0.99
2-Desoxy-D-glycero-pentos-3-ulose	-0.053	-0.21	0.11	0.51	0.99
O-Phosphoethanolamine	0.053	-0.11	0.21	0.52	0.99
Malic acid	-0.053	-0.21	0.11	0.52	0.99
Glyceryl-glycoside	-0.052	-0.21	0.11	0.52	0.99
Parabanic acid	0.052	-0.11	0.21	0.52	0.99
D-(-)-Lyxofuranose	0.052	-0.11	0.21	0.53	0.99
N-Acetylneuraminate	-0.051	-0.21	0.11	0.53	0.99
β-D-Glucopyranose	0.052	-0.11	0.21	0.53	0.99
D-Galactose (1Z)	-0.050	-0.21	0.11	0.54	0.99
D-(+)-Galacturonic acid	0.051	-0.11	0.21	0.54	0.99
Hypoxanthine	0.048	-0.11	0.21	0.56	0.99
1,3-Propanediol	0.046	-0.11	0.21	0.57	0.99
Phosphoric acid	0.045	-0.12	0.21	0.58	0.99
Nonacosane	-0.045	-0.21	0.12	0.59	0.99
Valine-d8	0.044	-0.12	0.20	0.59	0.99
Acetoacetic acid	0.045	-0.12	0.21	0.59	0.99
Eicosapentaenoic acid	-0.043	-0.20	0.12	0.60	0.99
Decenoyl-ACP	-0.043	-0.20	0.12	0.60	0.99
Isoleucine	0.041	-0.12	0.20	0.61	0.99
D-(-)-Rhamnose	-0.041	-0.20	0.12	0.62	0.99
4-amino-3-hydroxyButanoic acid	-0.040	-0.20	0.12	0.62	0.99
Dihydrouracil	-0.039	-0.20	0.12	0.63	0.99
D-Arabitol	0.039	-0.12	0.20	0.63	0.99
L-Norleucine	0.038	-0.12	0.20	0.64	0.99
Ornithine	-0.037	-0.20	0.12	0.64	0.99
1,6-Anhydroglucose	-0.037	-0.20	0.12	0.64	0.99
D-Glucose 6-phosphate	-0.037	-0.20	0.13	0.66	0.99
Phenylalanine	0.034	-0.13	0.19	0.67	0.99
Tryptophan	0.035	-0.13	0.20	0.67	0.99
D-Glucarate	-0.033	-0.19	0.13	0.68	0.99
Methionine	0.033	-0.13	0.19	0.68	0.99
Succinic acid-d4	0.032	-0.13	0.19	0.69	0.99
Oxalic acid	-0.032	-0.19	0.13	0.70	0.99
4,4-Dibromooctafluorobiphenyl	0.031	-0.13	0.19	0.70	0.99
Proline	-0.031	-0.19	0.13	0.71	0.99
3-Hydroxyicosatetraenoyl-CoA	-0.030	-0.19	0.13	0.72	0.99
Ribonic acid	-0.029	-0.19	0.13	0.72	0.99
1,5-Anhydrohexitol	-0.029	-0.19	0.13	0.72	0.99
L-Histidine	0.029	-0.13	0.19	0.72	0.99
3-Indolepropionic acid	-0.029	-0.19	0.13	0.72	0.99
Ethanolamine	-0.029	-0.19	0.13	0.73	0.99

Glyceric acid	-0.028	-0.19	0.13	0.73	0.99
Leucine	0.028	-0.13	0.19	0.73	0.99
Glycine	-0.028	-0.19	0.13	0.73	0.99
2-Keto-3-methylpentanoic acid	-0.028	-0.19	0.14	0.74	0.99
3-Pyridinol	-0.026	-0.19	0.14	0.75	0.99
Valine	-0.025	-0.18	0.13	0.75	0.99
Triethylene glycol	-0.025	-0.19	0.13	0.76	0.99
n-Propylamine	-0.025	-0.19	0.13	0.76	0.99
L-Threonic acid	-0.024	-0.18	0.13	0.76	0.99
Putrescine	-0.024	-0.18	0.14	0.77	0.99
Tyrosine	-0.023	-0.18	0.14	0.77	0.99
Benzoic acid	0.023	-0.14	0.19	0.78	0.99
Myo-Inositol	-0.023	-0.18	0.14	0.78	0.99
Hydroquinone	-0.022	-0.18	0.14	0.79	0.99
Urea	-0.022	-0.18	0.14	0.79	0.99
3-Indoleacetic acid	-0.021	-0.18	0.14	0.79	0.99
D-(-)-Tagatofuranose	-0.021	-0.18	0.14	0.80	0.99
Hexadecanoic acid. butyl	-0.021	-0.18	0.14	0.80	0.99
Phenoxyacetic acid	-0.020	-0.18	0.14	0.80	0.99
2-Hydroxybutyric acid	-0.019	-0.18	0.14	0.81	0.99
Lysine	0.017	-0.14	0.18	0.83	0.99
Heptadecanoic acid-d33	0.017	-0.14	0.18	0.84	0.99
Sorbitol	-0.015	-0.18	0.15	0.86	0.99
L-2-Aminobutyric acid	-0.014	-0.17	0.15	0.86	0.99
Isobutylamine	-0.014	-0.18	0.15	0.86	0.99
2-hydroxypropanoic acid	0.014	-0.15	0.17	0.87	0.99
α-D-(-)-Tagatopyran	-0.014	-0.18	0.15	0.87	0.99
N-Methyl-DL-Alanine	-0.012	-0.17	0.15	0.88	0.99
Maltose	-0.012	-0.17	0.15	0.88	0.99
L-Arginine	0.011	-0.15	0.17	0.89	0.99
L-α-Aminobutyric acid	-0.011	-0.17	0.15	0.90	0.99
L-(-)-Sorbose	-0.011	-0.17	0.15	0.90	0.99
D-Fucitol	-0.010	-0.17	0.15	0.90	0.99
meso-Erythritol	-0.010	-0.17	0.15	0.90	0.99
Threonine	-0.009	-0.17	0.15	0.92	0.99
Citric acid	0.008	-0.15	0.17	0.92	0.99
d-Glucose	0.008	-0.15	0.17	0.92	0.99
Glycolic acid	0.007	-0.15	0.17	0.93	0.99
Succinic acid	0.006	-0.15	0.17	0.94	0.99
Quininic acid	0.005	-0.15	0.16	0.95	0.99
Glycerol	-0.005	-0.17	0.16	0.95	0.99
L-(-)-Arabitol	0.005	-0.15	0.16	0.96	0.99
Alanine	-0.005	-0.16	0.16	0.96	0.99
4-Methylvaleric acid	-0.004	-0.16	0.16	0.96	0.99
D-(+)-Xylose	0.004	-0.16	0.16	0.96	0.99
L-Norvaline	-0.004	-0.16	0.16	0.96	0.99
Serine	-0.004	-0.16	0.16	0.96	0.99
D-Gluconic acid	0.002	-0.16	0.16	0.98	0.995
Glutamine	0.001	-0.16	0.16	0.99	0.998
Docosahexaenoic acid	-0.001	-0.16	0.16	0.99	0.998
Phosphoric acid, monomethyl ester	-0.0002	-0.16	0.16	1.00	0.998
